# TaPP2C-a5 fine-tunes wheat seed dormancy and germination with a Triticeae-specific, alternatively spliced transcript

**DOI:** 10.1016/j.jare.2025.05.007

**Published:** 2025-05-08

**Authors:** Qian Zhang, Xiaofen Yu, Ya’nan Wu, Ruibin Wang, Yufan Zhang, Fu Shi, Hongyan Zhao, Puju Yu, Yuesheng Wang, Mingjie Chen, Junli Chang, Yin Li, Guangyuan He, Guangxiao Yang

**Affiliations:** a The Genetic Engineering International Cooperation Base of Chinese Ministry of Science and Technology, Key Laboratory of Molecular Biophysics of Chinese Ministry of Education, College of Life Science and Technology, Huazhong University of Science and Technology, Wuhan 430074, China; b Key Laboratory of Plant Germplasm Enhancement and Specialty Agriculture, Wuhan Botanical Garden, Innovative Academy of Seed Design, Chinese Academy of Sciences, Wuhan 430074, China

**Keywords:** Clade A *PP2Cs*, Alternative splicing, ABA signalling, Seed dormancy and germination, Drought response, Wheat

## Abstract

**Introduction:**

The sessile plants often experience environmental conditions not ideal for growth, and therefore have evolved strategies to survive and adapt to stress conditions. Abscisic acid (ABA) regulates plant development and abiotic stress response. Clade A type 2C protein phosphatases (PP2Cs), act as co-receptors of ABA, negatively regulate ABA signalling. However, the biological function and detailed molecular mechanism of clade A *PP2Cs* in ABA signalling pathway remain to be elucidated in wheat.

**Objectives:**

To analyze the mechanisms of stress response and development mediated by ABA signal precisely regulated by TaPP2C-a5 at the post-transcriptional level in wheat, providing candidate genes for wheat improvement.

**Methods:**

Based on our previous results of *TaPP2Cs* gene family analysis, the function and detailed regulation mechanisms of *TaPP2C-a5* gene in seed dormancy and germination as well as drought response mediated by ABA signaling pathway were explored through reverse genetics technology.

**Results:**

We found that class A *TaPP2C-a5* underwent alternative splicing (AS) to produce two transcripts encoding TaPP2C-a5.1 and TaPP2C-a5.2, respectively. Both *TaPP2C-a5.1* and *TaPP2C-a5.2* were highly expressed in mature seeds, and were upregulated by exogenous ABA in seedlings. Overexpression of *TaPP2C-a5.1* and *TaPP2C-a5.2* coordinately negatively regulated seed dormancy and ABA-mediated seed germination as well as post-germination developmental arrest in wheat. *TaPP2C-a5.1* negatively regulated drought stress response, while *TaPP2C-a5.2* did not participate in drought stress response. The homologous genes of *TaPP2C-a5* underwent the same AS as *TaPP2C-a5* in tetraploid wheat, but not in rice.

**Conclusion:**

Our results revealed that *TaPP2C-a5* gene underwent AS and was involved in the regulation of seed dormancy and germination, as well as drought stress response mediated by the ABA signaling at the post-transcriptional level. Our work not only provide a potential target gene to improve PHS resistance, but also emphasize alternative splicing as a strategy with evolution contexts to fine-tune ABA signaling and its involvement in certain biological process.

## Highlights

• Clade A *TaPP2C-a5* gene undergoes alternative splicing to produce two transcripts encoding TaPP2C-a5.1 and TaPP2C-a5.2, respectively, in hexaploid wheat.
• The *TaPP2C-a5* alternative splicing exists in the Triticeae species but not in rice.
• TaPP2C-a5.1 and TaPP2C-a5.2 coordinately negatively regulate seed dormancy level and ABA-mediated seed germination as well as post-germination developmental arrest in wheat.
• TaPP2C-a5.1 negatively regulates drought stress response, while TaPP2C-a5.2 does not participate in drought stress response in wheat.
• We emphasize that the manipulation of *TaPP2C-a5.2* could be a promising strategy to regulate PHS resistance via fine-tuning the ABA signaling while not bringing obvious negative effects on drought-stress tolerance or plant growth.

## Graphical abstract

**Figure:**
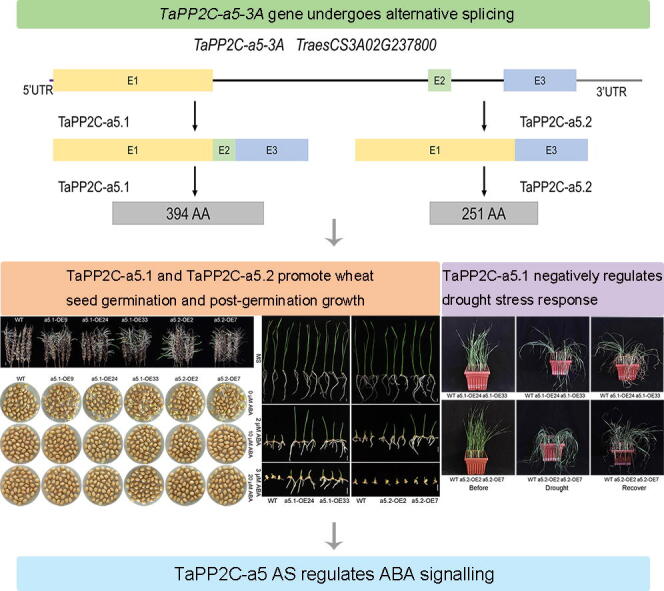

## Introduction

Owing to the sessile growth nature, plants are constantly challenged by variable environmental conditions, which seriously threaten their survival and productivity. Therefore, plants have evolved a series of complex and elaborate signaling mechanisms to respond to stress conditions and dynamically monitor metabolism and regulate growth and development [1]. Under stress, plants actively repress their growth to adapt to stress conditions, which can be achieved through stress-sensing and signaling pathways. This growth inhibition during stress is known as the stress-growth trade-off [2]. Currently, the trade-off between growth and stress resistance could be explained in two aspects. First, under stress, the energy and resource availability is limited. Accordingly, plants have to restrain the resources and energy use for growth and re-allocate them to stress response. Secondly, a number of phytohormonal and signaling transduction pathways have evolved to be sensitive to mild stresses and actively slow the growth rate, allowing plants to prepare for the possible more severe stresses in the future [2,3]. In line with this, overexpressions of the master regulators of abiotic stress responses could improve stress resistance while severely compromising growth.

During stress response and adaptation, the phytohormone abscisic acid (ABA) and its downstream signaling pathway play a crucial role [2,[4], [5], [6]]. In *Arabidopsis thaliana*, the core molecular mechanisms underlying ABA sensing and signal transduction have been well-established [[7], [8], [9]]. Under normal conditions, clade A PP2Cs interact with subclass III sucrose nonfermenting1-related protein kinase 2 s (e.g., SnRK2.2/2.3/2.6) and inhibit their kinase activity through dephosphorylation to turn off ABA signalling [7,[10], [11], [12]]. Under abiotic stress conditions or specific developmental signals, the plants rapidly accumulate a large amount of ABA. ABA is perceived by ABA receptors Pyrabactin resistance 1 (PYR1)/PYR1-like protein (PYL)/regulatory component of ABA receptor (RCAR) (hereafter referred to as PYL) [[13], [14], [15], [16]], which promote the interactions of ABA-PYLs with clade A PP2Cs, inhibit the phosphatase activity of PP2Cs, and release the kinase activity of SnRK2s from PP2Cs-SnRK2s complexes [4,17]. Activated SnRK2s then phosphorylate and activate downstream stress responsive proteins such as ion channels [18], and the ABA-responsive element binding factors (AREB/ABFs; such as ABI5) [19,20] to activate ABA signaling pathway mediating stress response and seed dormancy. Studies on other crop species support that the ABA signaling pathway is largely conserved among species [[21], [22], [23]].

Because of the central role of ABA in stress regulation, genes encoding ABA metabolic enzymes and core signaling components have been considered as the targets of genetic improvement towards increasing stress resistance. However, studies have demonstrated that manipulation of these ABA-core signaling genes, especially through ubiquitous overexpression or knock-out, often leads to compromised growth in plants. For example, mutant of ABA biosynthesis (*aba1*) of rice had weaker growth and a smaller biomass amount than those of the wildtype [24]. Multigene knockouts of *AtPYLs* exhibited significantly impaired ABA-induced stomatal closure and severe plant growth defects [25]. The *snrk2.2/2.3/2.6* mutant lost water extremely fast and was severely impaired in vegetative and reproductive growth [10,26]. In Arabidopsis, nine clade A PP2Cs were identified as co-receptors of ABA, and negatively regulated ABA signalling [11,12,[27], [28], [29], [30], [31]]. The *hab1-1abi1-2pp2ca-1* showed an extreme response to exogenous ABA, impaired growth and partial constitutive response to endogenous ABA [27]. Constitutive overexpression of *OsABI5*, one of the master ABA-responsive TFs and a negative regulator of salt tolerance, promoted growth inhibition, while repressing *OsABI5* expression *via* the antisense transgenic approach is associated with compromised fertility [32].

Besides the critical role in stress response and regulation, ABA signaling is widely involved in regulating various developmental processes, such as seed maturation, dormancy, germination, vegetative growth and senescence [5,6,33,34]. Therefore, molecular mechanisms that de-couple the ABA-associated trade-off between stress resistance and growth regulation should exist in plants. Identifying these molecular mechanisms in major crops could benefit the utilization of ABA signaling and candidate genes to improve developmental controls and enhance abiotic stress resistance.

In the present study, we chose seed dormancy and germination as the developmental process of interest and found that TaPP2C-a5 participated in the ABA signaling during seed maturation and germination in wheat. Interestingly, the *TaPP2C-a5* gene produced two transcripts through alternative splicing: one encoded an intact PP2C protein (namely TaPP2C-a5.1), while the other (namely TaPP2C-a5.2) encoded a protein that lacks the complete PP2C domain in hexaploid wheat. Interestingly, TaPP2C-a5.2 was Triticeae specific, which was not detected in rice. We also provided evidence that TaPP2C-a5.1 and TaPP2C-a5.2 coordinately negatively regulated seed dormancy levels and ABA-mediated seed germination as well as post-germination developmental arrest in wheat. In addition, TaPP2C-a5.1 negatively regulated drought stress response, while TaPP2C-a5.2 did not have obvious negative effects on drought-stress resistance. Therefore, specific increase expression of *TaPP2C-a5.2* during seed maturation stages may provide a novel strategy to fine-tune seed dormancy and germination in wheat while the strength of ABA signaling and responses remain unaffected during other developmental stages.

## Materials and methods

### Plant materials and growth conditions

The hexaploid wheat (*Triticum aestivum* L.) cultivar L88-31 [35] used for gene cloning, expression analyses and genetic transformation was grown in the experimental field of Huazhong University of Science and Technology, Wuhan, China. The *Nicotiana benthamiana* plants used for subcellular localization and bimolecular fluorescence complementation (BiFC) assays were grown in the pots filled with a mixture of soil:vermiculite (3:1) under greenhouse (16 h light/8h dark) conditions at 22 °C. Wheat seeds were sterilized with 75 % ethanol solution for 1 min followed by 0.1 % HgCl_2_ solution treatment for 8 min, and washed with sterile water at least four times.

### Total RNA extraction and qRT-PCR analysis

Young roots, stems, leaves from three-leaf-stage wheat seedlings; mature roots, stems, leaves, flag leaves, stamens from flowering stage of wheat plants; palea, lemma, caryopsis, embryo, endosperm from mature wheat plants at 20 days post anthesis (dpa) and grains at different dpa were collected to perform tissue-specific expression analysis. Two-week-old seedlings were steeped in the solution with or without 100 μM ABA. Then the leaves and roots from seedlings were collected at 0, 1, 3, 6, 12, and 24 h to perform expression analysis. The mature seeds were imbibed for 0, 3, 6, 12, 24, 36, and 48 h in the solution with or without 30 μM ABA, and then were collected to perform expression analysis. All samples were immediately frozen by liquid nitrogen and then stored at −80 °C for further analyses.

Total RNA was extracted using a Plant Total RNA Extraction Kit (Zomanbio, Beijing, China). Then the cDNA was produced by a HiScript III All-in-one RT SuperMix Perfect for qPCR (Vazyme, Beijing, China). The qRT-PCR was conducted in a CFX96 Connect Real-Time PCR Detection System (Bio-Rad, Hercules, CA, USA) using the AceQ qPCR SYBR Green Master Mix (Vazyme, Nanjing, China). The *TaActin* gene (Chinese Spring RefSeqv1.1 TraesCS1A02G274400) was used as the reference gene. Three biological replicates were performed for each experiment, and the relative gene expression levels were analysed using the comparative 2^−ΔΔCT^ method [36]. All primers used for gene expression were listed in Table S1.

### Gene cloning

The cDNA sequence of *TaPP2C-a5-3A* gene (Chinese Spring RefSeqv1.1 TraesCS3A02G237800) was amplified and the templates were synthesized from the total RNA isolated samples from the hexaploid wheat seedlings and different stages of developing grains. The homologous genes of *TaPP2C-a5-3A* in different plant species were obtained from Triticeae Gene Tribe (TGT) database (https://wheat.cau.edu.cn/TGT/). The partial coding DNA sequence (CDS) regions of *TaPP2C-a5*, *TdPP2C-a5* and *OsPP2C08* were amplified, and the templates were synthesized from the total RNA isolated from hexaploid wheat grains (*cv.* L88-31 and *cv.* Chinese Spring), tetraploid durum wheat grains (*cv*. Luna) and rice grains (*cv*. Nipponbare), respectively. The PCR products were analyzed by agarose gel electrophoresis (1 % and 2 %), and Sanger sequenced (Augct Biotech, Wuhan, China) to confirm the sequences. All the specific primers were listed in Table S1.

### Yeast two-hybrid (Y2H) assay

The pGBKT7-*TaDOG1Ls*, pGBKT7-*TaPYLs* and pGBKT7-*TaSnRK2s* plasmids were constructed from our laboratory [[37], [38], [39]]. The coding regions of *TaPP2C-a5.1*, *TaPP2C-a5.2*, *TaPP2C-a5-E1E2* and *TaPP2C-a5-E2E3* were fused into pGADT7 vectors. All cloning primers were listed in Table S1.

Y2H assay was performed according to the user manual of the MatchMaker^TM^ Gold Yeast Two-Hybrid system (Clontech, USA). The pGADT7-*TaPP2C-a5s* and pGBKT7-*TaDOG1Ls*, pGBKT7-*TaPYLs*, pGBKT7-*TaSnRK2s* were co-transformed into the yeast strain AH109. Interaction between SV40-T and p53 was used as a positive control, and the interaction between SV40-T and Lamin-C was used as a negative control, respectively. The positive transformants were selected by double-dropout medium (SD/-Trp-Leu, DDO), then examined by transferring to triple-dropout medium (SD/-His-Trp-Leu, TDO) with or without 10 μM ABA, and to quadruple-dropout medium (SD/-Ade-His-Trp-Leu, QDO) with or without 10 μM ABA.

### Protein subcellular localization of TaPP2C-a5 and BiFC assays

For protein subcellular localization assay, the coding regions of *TaPP2C-a5.1* and *TaPP2C-a5.2* were fused into the pBI121-*GFP* vectors, respectively. The pBI121-*GFP* (control vector), pBI121-*TaPP2C-a5.1-GFP* and pBI121-*TaPP2C-a5.2-GFP* were transformed into *Agrobacterium tumefaciens* strain EHA105, respectively. Then positive transformants were cultured and injected into the leaf epidermal cells of 4-week-old tobacco. For BiFC assay, the coding regions of *TaPP2C-a5.1* and *TaPP2C-a5.2* were fused into SpYNE vectors, respectively. The coding regions of *TaDOG1L1* and *TaSnRK2.8* were fused into SpYCE vectors. SpYCE-*TaDOG1L1*/*TaSnRK2.8* and SpYNE-*TaPP2C-a5.1*/*TaPP2C-a5.2* were transformed into *A. tumefaciens* strain EHA105. The leaf epidermal cells of 4-week-old tobacco were co-infiltrated with mixtures of an equal amount of SpYCE/SpYNE culture. After 48 h, GFP or YFP signal was detected using a fluorescence microscope (80i, Nikon, Japan). Nuclei were stained by the 4′,6-diamidino-2-phenylindole (DAPI) dye. The used primers were listed in Table S1.

### Generation and confirmation of transgenic wheat

To study the phenotypic effects of *TaPP2C-a5.1* and *TaPP2C-a5.2* overexpressing in wheat, the CDSs of *TaPP2C-a5.1* and *TaPP2C-a5.2* fused with 3 × HA tag were amplified and fused into the pAHC25 vectors, respectively, to drive ubiquitously high expression of *TaPP2C-a5.1* and *TaPP2C-a5.2* by using the *Zea mays ubiquitin1* promoter. The recombinant constructs were transformed into immature embryos of wheat *cv*. L88-31 by particle bombardment method [40]. The primers used to detect the positive transgenic plants were listed in Table S1. The transcript levels of the *TaPP2C-a5.1* and *TaPP2C-a5.2* genes in independent transgenic wheat lines were analyzed by qRT-PCR, respectively. The T_4_ generations of the *TaPP2C-a5.1* and *TaPP2C-a5.2* overexpressing lines of wheat were used in the present study.

### Pre-harvest sprouting and germination assay of transgenic wheat

For pre-harvest sprouting (PHS) test, the spikes of the WT, *TaPP2C-a5.1* and *TaPP2C-a5.2* transgenic lines were harvested at physiological maturity (as characterized by the loss of green color on the spike). The spikes were immersed in distilled water for three hours and then bundled with wet gauze and placed in plastic bags with 90 % humidity before they were incubated in a moist chamber at 22 °C for five days. Germinated and nongerminated seeds were counted in each spike and the percentage of visible sprouted kernels (PVSKs) were determined by using 20 randomized choosing spikes for each wheat line [41]. The germinated seeds were collected to analyze the expression levels of ABA responsive, biosynthesis and catabolism genes. For seed germination assay, about 50 seeds of WT, *TaPP2C-a5.1* and *TaPP2C-a5.2* transgenic lines were sterilized and sown on sterile filter paper in Petri dishes containing various concentrations of ABA (0, 10, or 20 μM ABA). The Petri dishes were subsequently transferred to a growth chamber at 22 °C for seven days. Visible radicle protrusion was considered as seed germination. Three independent biological replicates were conducted for this analysis.

### Seedling growth assays of transgenic wheat

To unify their germination, surface sterilised seeds of WT, *TaPP2C-a5.1* and *TaPP2C-a5.2* transgenic wheat were sown on MS plates and subsequently were stratified at 4 °C for 48 h in the dark before placing plates in the growth chamber at 22 °C. After 24 h of incubation in the growth chamber, uniformly germinated seeds were transferred to glass bottles containing MS medium with or without various concentrations of ABA (0, 2, or 3 μM ABA). After 10 d incubation, the root and shoot lengths of plants were measured. Three independent biological replicates were conducted for this analysis.

### Drought stress to transgenic wheat

To analyze the effect of drought stress on transgenic wheat, uniformly germinated seeds of WT, *TaPP2C-a5.1* and *TaPP2C-a5.2* transgenic lines were cultivated in soil for 3 weeks with normal conditions. The three-leaf-stage seedlings were subjected to about 25 days of drought stress conditions (no water supply), and then were supplied with water for 2 d to allow recovery. Then the surviving plants in each line were counted. Three independent biological replicates were performed for this analysis. At least 16 seedlings were observed in each wheat line in each replicate. Survival rate = number of surviving plants/total number of plants.

### RNA-seq expression analysis

The RNA-seq expression data of hexaploid wheat across five tissues at different developmental stages (Root_Z10, Z13 and Z39; Stem_Z30, Z32 and Z65; Leaf_Z10, Z23 and Z71; Spike_Z32, Z39 and Z65; Grain_Z71, Z75 and Z85) [42], developing wheat grain (different cell types at 10, 20 or 30 dpa) [43], embryo and endosperm in developing wheat grain (at 14 or 25 dpa) [44], and wheat abiotic stress (drought stress) [45,46] were retrieved from the GeneExpression of WheatOmics database (https://202.194.139.32/) to examine the expression profiles of clade A *TaPP2C* genes. The expression heat maps were visualized using TBtools software. The gene expression levels were quantified using log_2_(transcripts per million (TPM) + 1) [47].

### Sequence analysis

The protein sequences of class A PP2Cs were downloaded from the Ensembl Plants database (https://plants.ensembl.org/index.html). Multiple sequence alignments of class A PP2Cs were performed with the MAFFT method.

### Statistical analysis

All experiments were performed with three biological replicates in the present study. The statistical significant differences were calculated with the SPSS software by using Student’s *t*-test (* *P* < 0.05, ** *P* < 0.01, *** *P* < 0.001) or ANOVA with the *post-hoc* statistical test (*P* < 0.05).

## Results

### Identification of an evolutionarily conserved alternative splicing (AS) event of *TaPP2C-a5* in Triticeae

A total of 15 clade A *PP2C* genes were identified in wheat, which were divided into ABI1 (*TaPP2C-a1* to *TaPP2C-a4*) and AHG1 (*TaPP2C-a5* to *TaPP2C-a15*) subfamilies [39]. Based on published RNA-seq data, members of *ABI1* subfamily (except for *TaPP2C-a2-1B*, which is highly expressed in seeds) are expressed in various tissues, highly expressed in leaves, and expressed in seeds at very low level. In contrast, members of *AHG1* subfamily show different expression patterns: *TaPP2C-a7/a8/a9* are highly expressed in both leaves and seeds, especially in leaves; *TaPP2C-a10* is highly expressed in both seeds and leaves; *TaPP2C-a5/a6* are preferentially expressed in seeds (Fig. S1A). These are similar to the expression patterns of clade A *AtPP2Cs* [48], suggesting that there may be functional differentiation between the ABI1 and AHG1 subfamilies in wheat. In addition, *TaPP2C-a5/a7* are highly expressed in the aleurone layer (AL) of grains, while *TaPP2C-a3* is highly expressed in the transfer cell (TC) of grains (Fig. S1B). Meanwhile, the expression levels of *TaPP2C-a5/a7* are relatively high in embryos at different dpa, but very low in endosperm. In contrast, the expression levels of *TaPP2C-a3* are relatively high in both embryos and endosperm at the 25 dpa (Fig. S1C). In addition, *TaPP2C-a3/a5/a7* are induced by drought stress (Fig. S1D-E). These results suggest that the ABI1 subfamily members may primarily play a role in drought stress response, while the AHG1 subfamily members may primarily play a role in seed dormancy and germination as well as drought stress response.

**Fig. 1:**
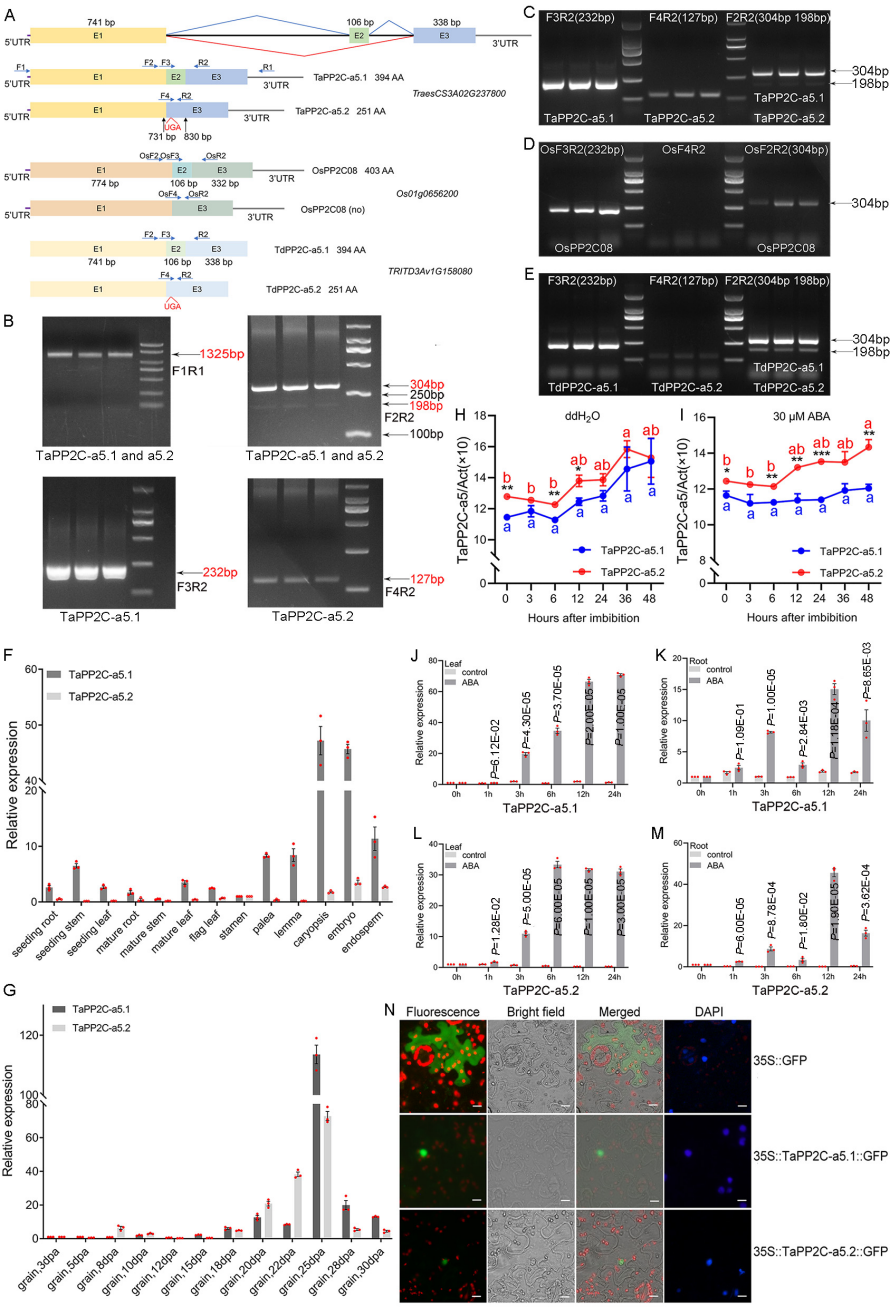
Identification and characterization of an alternative splicing event of *TaPP2C-a5* possibly conserved between the Triticeae species. (**A**) Schematic diagram outlining the organization of *TaPP2C-a5*, *OsPP2C08* and *TdPP2C-a5*. ‘E’ indicates exons, the black line indicates introns. (**B**) Images of agarose gels of PCR amplification products of *TaPP2C-a5* cDNA (upper left, 1 % agarose gel) and partial coding sequences (other three, 2 % agarose gel). Total RNA was isolated from hexaploid wheat *cv*. L88-31 seedlings and grains. Four primer pairs (F1R1, F2R2, F3R2 and F4R2) were used to detect *TaPP2C-a5.1* and *TaPP2C-a5.2*. (**C)** Images of 2 % agarose gels of PCR amplification products of *TaPP2C-a5* partial coding sequences. Total RNA was isolated from hexaploid wheat *cv*. Chinese Spring seeds. Three primer pairs (F2R2, F3R2 and F4R2) were used to detect the *TaPP2C-a5.1* and *TaPP2C-a5.2*. (**D**) Images of 2 % agarose gels of PCR amplification products of *OsPP2C08* partial coding sequences. Total RNA was isolated from rice *cv*. Nipponbare seeds. Three primer pairs (OsF2R2, OsF3R2 and OsF4R2) were used to detect the *OsPP2C08*. (**E**) Images of 2 % agarose gels of PCR amplification products of *TdPP2C-a5* partial coding sequences. Total RNA was isolated from tetraploid wheat *cv*. Luna seeds. Three primer pairs (F2R2, F3R2 and F4R2) were used to detect *TdPP2C-a5.1* and *TdPP2C-a5.2*. (**F**, **G**) Expression levels of *TaPP2C-a5.1* and *TaPP2C-a5.2* in wheat different tissues (**F**) and dpa grains (**G**) were quantified by qRT-PCR. (**H**, **I**) The dynamic expressions of *TaPP2C-a5.1* and *TaPP2C-a5.2* during seed imbibition time points without (**H**) or with 30 μM ABA (**I**) were quantified by qRT-PCR. Statistical differences of expression levels between the time points within the same transcript were calculated with ANOVA and were indicated with letters (*post-hoc* Tukey’s test, *P* < 0.05), while the statistical differences of expression levels between *TaPP2C-a5.1* and *TaPP2C-a5.2* for the same time point were determined by Student’s *t*-test and are indicated by asterisks (* *P* < 0.05, ** *P* < 0.01, *** *P* < 0.001). (**J**, **K**) The dynamic expressions of *TaPP2C-a5.1* in response to 100 μM ABA in seedling leaves (**J**) and roots (**K**) were quantified by qRT-PCR. (**L**, **M**) The dynamic expressions of *TaPP2C-a5.2* in response to 100 μM ABA in seedling leaves (**L**) and roots (**M**) were quantified by qRT-PCR. *TaActin* was used as the internal reference gene. The expression data were presented as means ± standard error (S.E.) for three biological replicates, with each replicate meaning the pooled sample of three plants (**F**, **G**, **J**, **K**, **L**, **M**) or six imbibed seeds (**H**, **I**). The averaged expression levels of three technical repeats per replicate were used for data analysis. The statistical differences of expression levels for the same time point between the control and ABA treated samples were calculated with Student’s *t*-test, with *P*-values indicated on the figures. (**N**) The subcellular localization analysis of TaPP2C-a5.1 and TaPP2C-a5.2. The GFP vector was transformed as control. The subcellular localization experiments were repeated three times with similar results, and representative images were presented.

To investigate the clade A *PP2Cs* with seed-specific expression patterns that are potentially involved in seed maturation and germination in wheat, we performed RT-PCR and cloned the transcripts of clade A *TaPP2C-a5* genes from the maturing and germinating seeds of wheat (*cv*. L88-31). Our results demonstrated that *TaPP2C-a5* had two transcripts (cloned with the primers F1 and R1, designated as *TaPP2C-a5.1* and *TaPP2C-a5.2*, respectively), one corresponding to the full-length transcript of *TaPP2C-a5* comprised of three exons, the other containing only the exon 1 and 3. In particular, the *TaPP2C-a5.2* transcript was predicted to encode a premature PP2C protein with a partial PP2C phosphatase domain (Fig. 1A-B; Fig. S2) (Table S2). Further, we designed a series of PCR primers and validated the two *TaPP2C-a5* transcripts in hexaploid wheat *cv*. Chinese Spring (Fig. 1C). Moreover, we used the exons 1 and 3 junction sequence to search the publicly available RNA-seq sets of common wheat (NCBI SRA database, accession data SRA: DRR003150.7093710.1) and successfully found RNA-seq read identically matched the junction, further supporting the presence of *TaPP2C-a5.2* transcript (Fig. 1A; Fig. S3A-B) (Table S2).

Triticeae species have evolved to obtain specific genomic features in comparison to other diploid Poaceae food crops (e.g., rice, sorghum), such as the recent burst of gene duplications (RBGDs) and retrotransposons-driven gene expansion [[49], [50], [51]]. Such dramatic differences in the genome content (i.e., duplicated genes, transposable elements) and the patterns of gene duplication events in hexaploid wheat and its tetraploid and diploid relatives are thought to be associated with the changes in growth habits (for example, grow in winter/spring versus in summer for wheat and rice, respectively) and the evolution of Triticeae species. As such, we selected rice as the outgroup species when analyzing the *TaPP2C-a5* alternative transcripts. We therefore investigated whether the *TaPP2C-a5.2* transcript could exist in other Triticeae species or the monocot model species rice. We experimentally validated the presence of both *TdPP2C-a5.1* and *TdPP2C-a5.2* transcripts in the tetraploid durum wheat (*Triticum turgidum*, *cv*. Luna [52]), whereas in rice only *OsPP2C-a5.1* (*OsPP2C08*), but not *OsPP2C-a5.2*, was detected (*cv*. Nipponbare) (Fig. 1A, 1D-E). For other Triticeae species such as barley (*Hordeum vulgare*), the *HORVU3Hr1G059170* gene may undergo AS to produce two transcripts as indicated in PacBio Iso-seq data [53], one of which is a full-length transcript and the other contains the first intron (Fig. S3C, S3F) (Table S3, S4). In addition, in PacBio Iso-seq data of *Aegilops comosa* [54], the *AeCom.PI551049.r1.3MG022460* gene may undergo AS to produce at least four transcripts, one of which is similar to *TaPP2C-a5.2* transcript (Fig. S3C, S3G) (Table S3, S5). Based on the PacBio Iso-Seq data sets and genome annotations, the homologous genes of *TaPP2C-a5* in *Sorghum bicolor* [55], *Zea mays*, *Brachypodium distachyon*, and *Setaria italica* [56] have only one full-length transcript, matching well with the structure of *TaPP2C-a5.1* (Fig. S3C, S3H-K) (Table S3). Our results indicate that AS of *TaPP2C-a5* could likely occur specifically in Triticeae species, which implies that the AS of clade A *PP2C* genes plays an important role in the evolution of wheat.

### *TaPP2C-a5*.2 is mainly expressed during the reproductive growth and induced by ABA

High-throughput transcriptome sequencing studies revealed that 95 %-100 % of multi-exon genes in human undergo AS to produce two or more alternative isoforms, while 60 %-70 % of genes occur AS in plants in a tissue-specific, developmental or signal transduction dependent manners [[57], [58], [59], [60], [61], [62], [63]]. AS mediates plant biological processes from growth and development to biotic and abiotic stress responses, such as, flowering time [64,65], circadian rhythms [66], disease resistance [67], and stress responses [68,69]. We reasoned that the TaPP2C-a5.2 might have a particular biological function owing to the evolutionary conservation of this AS event and a possible regulatory role. We then scrutinized the expression patterns of *TaPP2C-a5.1* and *TaPP2C-a5.2*, respectively, across different tissues and developmental stages of wheat, with a focus on grain developing stages ranging from the developing grains at 3 to 30 dpa (almost mature) (Fig. 1F-G). Interestingly, we found that *TaPP2C-a5.1* was expressed with an obvious tissue preference, highly expressed in reproductive organs (i.e., palea, lemma, embryo and endosperm), but at much lower expression levels in vegetative tissues. Across these tissues and developmental stages, *TaPP2C-a5.1* always exhibited much higher expression levels than those of the *TaPP2C-a5.2* (Fig. 1F). During wheat grain development, both *TaPP2C-a5.1* and *TaPP2C-a5.2* were up-regulated. In maturing grains (from 20 to 30 dpa), the expression of *TaPP2C-a5.2* was dramatically incresased to a level comparable to that of *TaPP2C-a5.1* (Fig. 1G). The high expression levels of *TaPP2C-a5.2* during maturing seeds, as well as the potential involvement of TaPP2C-a5 in ABA signaling, prompted us to hypothesize that TaPP2C-a5.2 could have a particular role in seed dormancy and germination. To this end, we quantified the expression levels of *TaPP2C-a5.1* and *TaPP2C-a5.2* during wheat seed germination with or without 30 μM ABA. Without the ABA treatment, *TaPP2C-a5.2* expression levels were slightly higher than those of *TaPP2C-a5.1* within the first 24 h of seed imbibition, while *TaPP2C-a5.2* was induced by ABA, showing higher expression levels compared with those of *TaPP2C-a5.1* (Fig. 1H-I), further supporting the hypothetical role of TaPP2C-a5.2 in seed dormancy and germination. In addition, the expression levels of *TdPP2C-a5.1* and *TdPP2C-a5.2* were detected during seed imbibition of tetraploid wheat *cv*. Luna with or without 30 μM ABA (Fig. S3D-E). Moreover, we investigated whether the *TaPP2C-a5.2* transcript could play a role in other ABA-involved developmental processes. We measured the expression levels of both *TaPP2C-a5.1* and *TaPP2C-a5.2* in the leaf and root tissues of wheat seedlings with or without the 100 μM ABA treatment. At the seedling stage, both *TaPP2C-a5.1* and *TaPP2C-a5.2* were up-regulated since 3 h of ABA treatment (Fig. 1J-M). Then, we transiently expressed the *TaPP2C-a5.1-GFP* and *TaPP2C-a5.2-GFP* in tobacco leaves by using their cDNA sequences to examine protein subcellular localization. Both TaPP2C-a5.1-GFP and TaPP2C-a5.2-GFP were expressed in tobacco leaves and the corresponding GFP-fusion proteins were localized in the nucleus (Fig. 1N), supporting that the *TaPP2C-a5.2* transcript can be translated and possibly exerts regulatory functions.

### TaPP2C-a5.1, but not TaPP2C-a5.2 interacts with TaDOG1L1

**Fig. 2:**
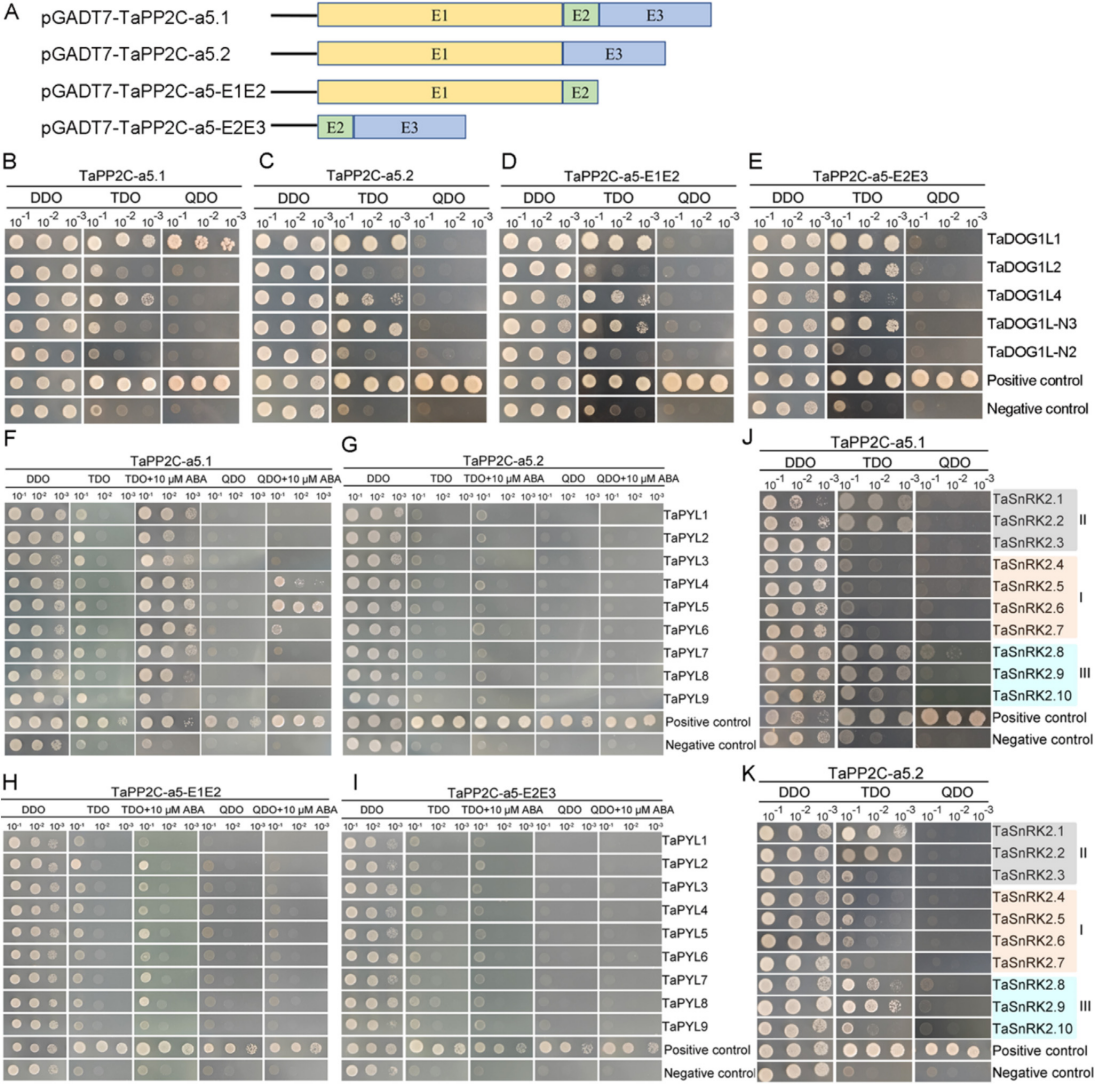
The Y2H analysis of TaPP2C-a5 and TaDOG1Ls, TaPYLs as well as TaSnRK2s. (**A**) Schematic diagram illustrates coding sequences of *TaPP2C-a5* were constructed into pGADT7 vector. (**B**, **C**, **D**, **E**) The interactions of pGADT7-TaPP2C-a5.1 (**B**), TaPP2C-a5.2 (**C**), TaPP2C-a5-E1E2 (**D**), TaPP2C-a5-E2E3 (**E**) and pGBKT7-TaDOG1Ls using Y2H assay. (**F**, **G**, **H**, **I**) The interactions of pGADT7-TaPP2C-a5.1 (**F**), TaPP2C-a5.2 (**G**), TaPP2C-a5-E1E2 (**H**), TaPP2C-a5-E2E3 (**I**) and pGBKT7-TaPYLs using Y2H assay. **(J**, **K)** The interactions of pGADT7-TaPP2C-a5.1 (**J**), TaPP2C-a5.2 (**K**) and pGBKT7-TaSnRK2s using Y2H assay. Positive transformants were cultured on selective medium DDO (SD/-Leu/-Trp), TDO (SD/-Trp-Leu-His) with or without 10 µM ABA, QDO (SD/-Trp-Leu-His-Ade) with or without 10 µM ABA. Interactions between SV40-T and p53 or Lamin-C were set as positive or negative control, respectively. Three independent experiments were performed with similar results, and representative images are presented.

We sought to confirm these potential functions at the protein level, because the results of subcellular localization and expression of TaPP2C-a5.1 and TaPP2C-a5.2 suggest their roles in seed dormancy and germination and potential regulation of ABA signaling. Since TaPP2C-a5.2 is encoded by AS of *TaPP2C-a5*, we artificially generated TaPP2C-a5-E1E2 and TaPP2C-a5-E2E3 cDNA to serve as the controls for Y2H assays (Fig. 2A). *DOG1* was a member of a small gene family unique to plants and was first identified as a quantitative trait locus (QTL) positively regulating seed dormancy in Arabidopsis [70,71]. The previous studies indicated that DOG1 interacted with AHG1 and AHG3 to control Arabidopsis seed dormancy. *AHG1* was seed-specifically expressed and showed the highest transcript levels in dry seeds among nine clade A *PP2Cs* [48,72]. For seed dormancy, we investigated the interactions between TaPP2C-a5 and TaDOG1 like proteins (i.e., TaDOG1L1, TaDOG1L2, TaDOG1L4, TaDOG1L-N2 and TaDOG1L-N3) [38]. TaPP2C-a5.1 could interact with TaDOG1L1, but not the other TaDOG1Ls (Fig. 2B). By contrast, neither TaPP2C-a5.2 nor the synthetic TaPP2C-a5-E1E2 and TaPP2C-a5-E2E3, could interact with any TaDOG1Ls (Fig. 2C-E**)**. Moreover, the BiFC assay results further confirmed that TaPP2C-a5.1, but not TaPP2C-a5.2 interacted with TaDOG1L1, and fluorescence signal was observed in the nucleus (Fig. S4). These results suggest that interaction of TaDOG1L1 and TaPP2C-a5 may require complete PP2C activity and TaPP2C-a5 may be involved in the regulation of wheat seed dormancy.

### TaPP2C-a5.1 and TaPP2C-a5.2 are involved in ABA signaling

In parallel, we examined the protein interactions between the TaPP2C-a5 and the core components in wheat ABA signaling pathway (i.e., TaPYLs and TaSnRK2), as the clade A PP2Cs act as negative regulators in the ABA signaling in plants [73]. In the absence of ABA, TaPP2C-a5.1, TaPP2C-a5.2, TaPP2C-a5-E1E2 and TaPP2C-a5-E2E3 did not interact with any TaPYLs. In the presence of 10 μM ABA, TaPP2C-a5.1 interacted with TaPYL4/5/6, while the other three did not interact with any TaPYLs (Fig. 2F-I). Moreover, both TaPP2C-a5.1 and TaPP2C-a5.2 interacted with class III TaSnRK2.8, but not any member of class I or II TaSnRK2s (Fig. 2J-K). Meanwhile, the BiFC assays further proved their interactions, and the interactions between TaSnRK2.8 and TaPP2C-a5.1 or TaPP2C-a5.2 were observed in the nucleus (Fig. S4). The crystal structure of PYLs-ABA-PP2Cs complex indicates that the PYLs-mediated “Gate-Latch-Lock” mechanism involves the active site and conserved Trp of PP2Cs. The highly stable PYLs-ABA-PP2Cs ternary complex inhibits the phosphatase activity of PP2Cs [[74], [75], [76], [77], [78], [79]]. Thus, the other three variants of TaPP2C-a5 may lack the phosphatase active site and conserved Trp necessary for interactions with TaPYLs (Fig. 2A; Fig. S2). The TaPP2C-a5.2 isoform, which may not be inhibited by the ABA-PYLs complex, could still potentially regulate kinase activity of class III TaSnRK2.8. These results suggest that TaPP2C-a5 may be involved in regulating the ABA signaling pathway in wheat by interacting with core components of ABA signaling.

### TaPP2C-a5.1 and TaPP2C-a5.2 coordinately regulate wheat seed dormancy and germination

**Fig. 3:**
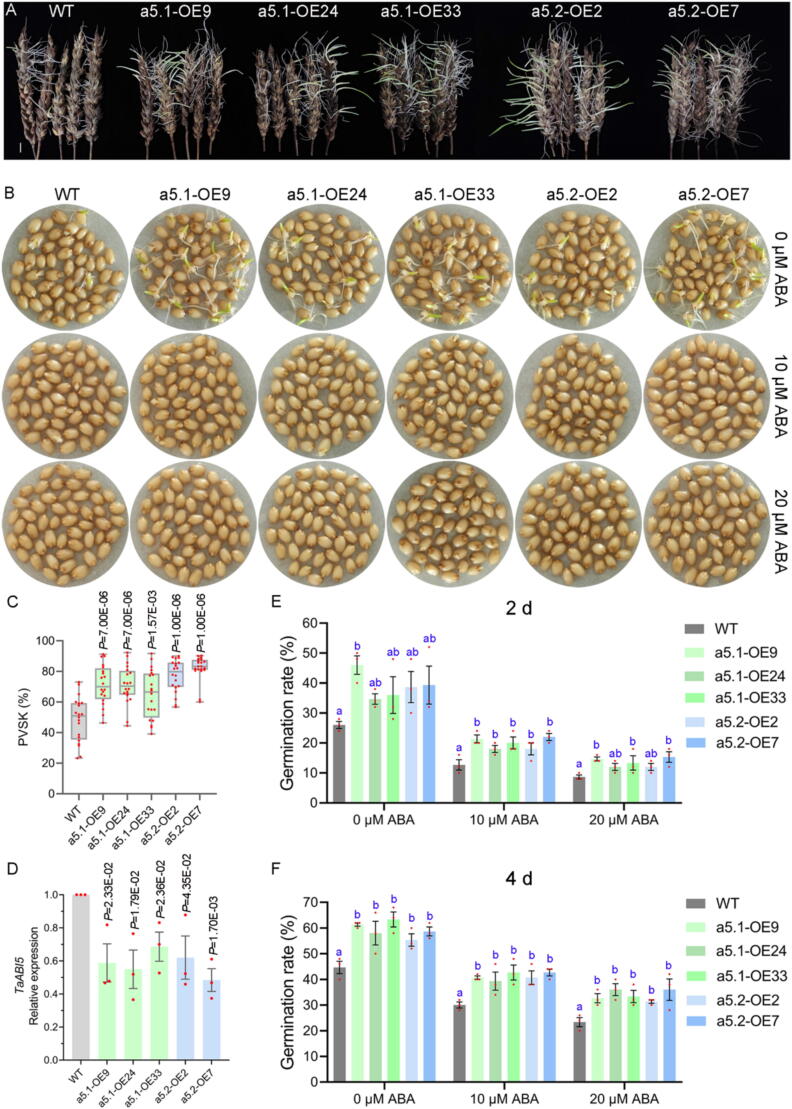
TaPP2C-a5.1 and TaPP2C-a5.2 modulate PHS and ABA responses in wheat. (**A**) Representative images showing the PHS phenotypes of the WT, *TaPP2C-a5.1* and *TaPP2C-a5.2* OE lines. Scale bar represents 1 cm. (**B**) Phenotypes of seeds germination of WT, *TaPP2C-a5.1* and *TaPP2C-a5.2* OE lines at 2 d after imbibition. The seeds were incubated in distilled water with 0, 10, or 20 μM ABA. (**C**) Box plots summarize the PVSKs from 20 spikes for each of WT, *TaPP2C-a5.1* and *TaPP2C-a5.2* OE lines. The experiment was performed with three replicates with each replicate containing at least six spikes per WT or OE line. (**D**) qRT-PCR analysis of *TaABI5* expression levels in seeds of PHS of WT, *TaPP2C-a5.1* and *TaPP2C-a5.2* OE lines. The experiment was performed with three replicates with each replicate containing the pooled sample of six germinating seeds per WT or OE line. The averaged expression levels of three technical repeats per replicate were used for data analysis. *P*-values indicate significant differences between OE lines and WT determined by Student’s *t*-test. (**E**, **F**) Seed germination tests of WT, *TaPP2C-a5.1* and TaPP2C-*a5.2* OE lines with the presence of 0, 10, or 20 µM ABA at 2 d (**E**) and 4 d (**F**), respectively. Data at 2 d or 4 d after seed imbibition are presented as means ± S.E. of three independent replicates. At least 50 seeds per WT or OE lines were used in each replicate. Statistical differences in germination rates were first calculated with two-way ANOVA considering the lines and ABA concentrations as the two factors (Table S6). To determine whether the OE lines and WT had different germination rates, the statistical differences were then calculated with one-way ANOVA for a given ABA concentration, with the *post-hoc* LSD test results indicated with letter presentation (*P* < 0.05).

To study the function of TaPP2C-a5.2, we generated transgenic wheat lines overexpressing *TaPP2C-a5.2* (namely a5.2-OE2 and OE7 in the donor cultivar L88-31). For comparison, we also obtained transgenic lines overexpressing *TaPP2C-a5.1* driven with the same *ubiquitin1* promoter (namely a5.1-OE9, OE24 and OE33) (Fig. S5A-H). The qRT-PCR analysis confirmed that the expression levels of *TaPP2C-a5.1* and *TaPP2C-a5.2* were higher in the corresponding *TaPP2C-a5.1* and *TaPP2C-a5.2* OE lines, respectively, than those in the WT (Fig. S5I-J). ABA is a major phytohormone that affects seed dormancy and germination, and when the seed dormancy level is too low, the PHS or vivipary phenotype could happen frequently [80]. We evaluated the PHS phenotypes for the *TaPP2C-a5.1* or *TaPP2C-a5.2* OE lines and the WT control. All *TaPP2C-a5.1* and *TaPP2C-a5.2* OE lines displayed more visible PHS phenotypes compared to WT (Fig. 3A). The percentage of visible sprouted kernels (PVSKs) for the three *TaPP2C-a5.1* OE lines (a5.1-OE9, OE24 and OE33) were 46.34 %-91.18 %, 46.88 %-92.31 %, and 39.13 %-91.67 %, respectively. The PVSKs for the two *TaPP2C-a5.2* OE lines (a5.2-OE2 and OE7) were 56.67 %-90 % and 60 %-90.32 %, respectively. Meanwhile, the PVSKs for WT were 23.33 %-73.08 % (Fig. 3C). The transcription factor ABI5 plays a crucial role in the regulation of seed dormancy and germination mediated by ABA signaling [81]. We quantified the expression level of the canonical ABA-responsive gene *TaABI5*. Indeed, the *TaABI5* expression levels were significantly lower in the seeds of both *TaPP2C-a5.1* and *TaPP2C-a5.2* OE lines than those in the WT seeds (Fig. 3D). The 9-*cis*-epoxycarotenoid dioxygenase (NCED) and ABA8′-hydroxylase (ABA8′OH) are key enzymes in ABA synthesis and catabolic pathways [4,82,83]. The expression levels of *TaNCEDs* and *TaABA8′OHs* were quantified in germinated seeds. The expression levels of *TaNCED1* and *TaNCED2* were significantly higher in both *TaPP2C-a5.1* and *TaPP2C-a5.2* OE lines than those of WT (Fig. S6A-B). Compared with WT, the expression levels of *TaABA8′OH1* were decreased, while the expression levels of *TaABA8′OH2* were increased in *TaPP2C-a5.1* and *TaPP2C-a5.2* OE lines (Fig. S6C-D). These results suggest that TaPP2C-a5 may affect ABA signaling and ABA metabolic pathways. Then, we analyzed the ABA response of *TaPP2C-a5* OE lines during seed germination. Compared with WT, both *TaPP2C-a5.1* OE lines (a5.1-OE9, OE24 and OE33) and *TaPP2C-a5.2* OE lines (a5.2-OE2 and OE7) exhibited accelerated seed germination in the absence of ABA. The 10 or 20 μM ABA treatment clearly suppressed the seed germination of WT, whereas the *TaPP2C-a5.1* and *TaPP2C-a5.2* OE lines exhibited ABA-insensitive inhibition in seed germination (Fig. 3B, 3E, 3F). During the time course of seed germination, the seed germination rates of the *TaPP2C-a5.1* and *TaPP2C-a5.2* OE lines were much higher than those of WT in the presence of 0, 10, or 20 μM ABA (Fig. 3E-F) (Table S6). These results demonstrate that both TaPP2C-a5.1 and TaPP2C-a5.2, as negative regulators of ABA signaling, promote seed germination.

### Results of *TaPP2C-a5.1* and *TaPP2C-a5.2* transgenic lines of wheat suggest differentially regulated ABA signaling during vegetative growth

**Fig. 4:**
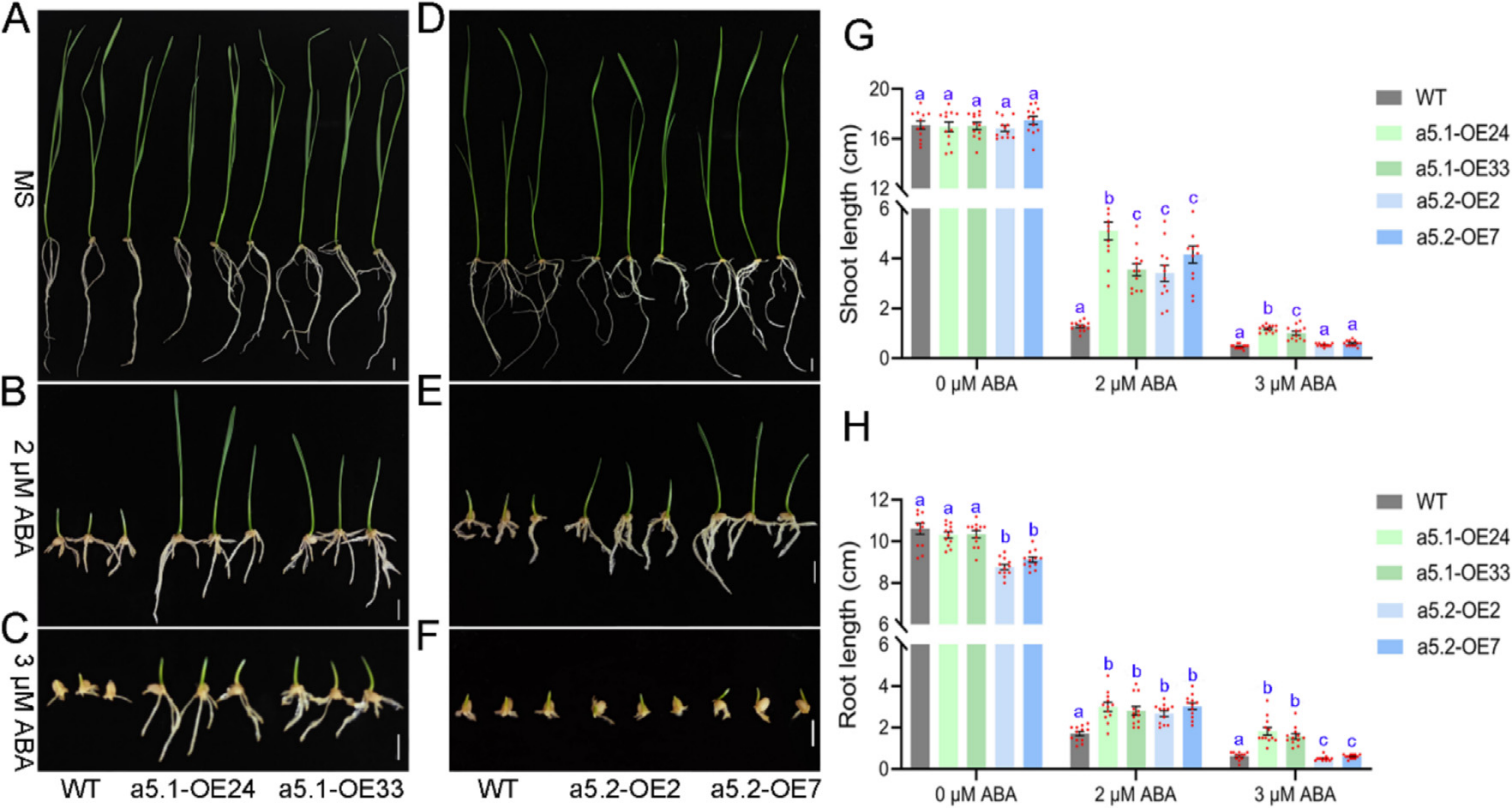
*TaPP2C-a5.1* and *TaPP2C-a5.2* overexpression in wheat promote seedling growth under ABA treatments. (**A**, **B**, **C**) Phenotypes of shoots and roots of WT and *TaPP2C-a5.1* OE lines treated with 0 (**A**), 2 (**B**), or 3 (**C**) µM ABA for 10 d. (**D**, **E**, **F**) Phenotypes of shoots and roots of WT and *TaPP2C-a5.2* OE lines treated with 0 (**D**), 2 (**E),** or 3 (**F**) µM ABA for 10 d. Scale bar represents 1 cm. Three independent experiments were performed with similar results, and representative images are presented. (**G**, **H**) Comparisons of the shoot (**G**) and root (**H**) lengths of *TaPP2C-a5.1*, *TaPP2C-a5.2* OE lines and WT. Shoot and root lengths were measured after uniformed germinated seeds of WT, *TaPP2C-a5*.1 and *TaPP2C-*a5.2 OE lines were transferred to MS medium containing 0, 2, or 3 µM ABA for ten days. The ABA-treatment experiment was performed in triplicates, and within each replicate four seedlings per WT or OE line (a total of 12 seedlings for three replicates) were used for meauring shoot and root length. Statistical differences in shoot or root length were first calculated with two-way ANOVA considering the lines and ABA concentrations as the two factors (Table S7). To determine whether the OE lines and WT had different germination rates, the statistical differences were then calculated with one-way ANOVA for a given ABA concentration, with the *post-hoc* LSD test results indicated with letter presentation (*P* < 0.05).

To further investigate the roles of TaPP2C-a5 in ABA signalling, we examined the early seedling development of *TaPP2C-a5.1* OE lines (a5.1-OE24 and OE33) and *TaPP2C-a5.2* OE lines (a5.2-OE2 and OE7) in response to exogenous ABA. Without ABA treatment, the shoot lengths of both *TaPP2C-a5.1* and *TaPP2C-a5.2* OE lines were similar to those of the WT, while root lengths of *TaPP2C-a5.2* OE lines were significantly lower than those of the WT, which were similar to those of *TaPP2C-a5.1* OE lines (Fig. 4A, 4D, 4G-H). The exogenous ABA applications inhibited shoots and roots elongation of WT, *TaPP2C-a5.1* and *TaPP2C-a5.2* OE lines. However, the inhibition of shoots and roots elongation in WT were greater than those of both *TaPP2C-a5.1* and *TaPP2C-a5.2* OE lines after 2 μM ABA treatment (Fig. 4B, 4E, 4G-H). The shoots and roots of *TaPP2C-a5.1* OE lines showed stronger ABA insensitivity than those of *TaPP2C-a5.2* OE lines. The seedling growth in *TaPP2C-a5.2* OE lines were almost completely suppressed after 3 μM ABA application, while *TaPP2C-a5.1* OE lines still grew and exhibited longer roots and shoots (Fig. 4C, 4F, 4G-H) (Table S7). These data suggest that *TaPP2C-a5* variants negatively regulate ABA signalling during early wheat seedlings development.

### Overexpression of *TaPP2C-a5.1* but not *TaPP2C-a5.2* negatively affects the drought tolerance in transgenic wheat

**Fig. 5:**
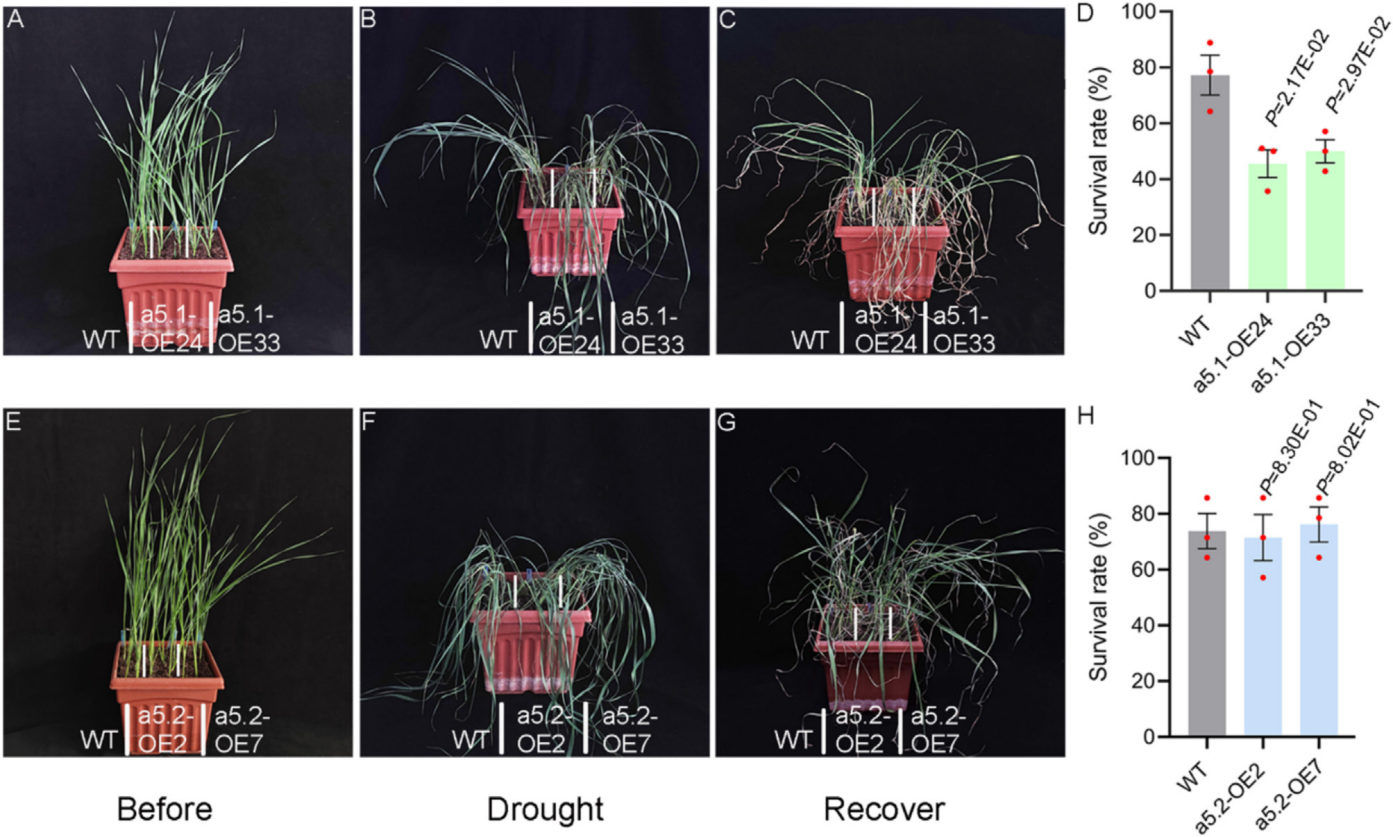
Overexpression of *TaPP2C-a5.1* not *TaPP2C-a5.2* negatively affects the drought tolerance of transgenic wheat. (**A**, **B**, **C**) Phenotypes of WT and *TaPP2C-a5.1* OE lines under drought stress. Two-week-old seedlings (**A**) grown in soil were treated by stopping irrigation for 20 d (**B**) and then rewatering for 2 d (**C**). (**D**) Survival rates of WT and *TaPP2C-a5.1* OE lines in response to drought stress. (**E**, **F**, **G**) Phenotypes of WT and *TaPP2C-a5.2* OE lines under drought stress. Two-week-old seedlings (**E**) grown in soil were treated by stopping irrigation for 24 d (**F**) and then rewatering for 2 d (**G**). (**H**) Survival rates of WT and *TaPP2C-a5.2* OE lines in response to drought stress. The drought experiments were performed with three replicates with similar results, and representative images are presented. In each replicate, at least 16 seedlings were used for the measurement of survival rate, with the data presented as means ± S.E. of three replicates. Statistical differences between OE lines and WT were calculated with Student’s *t*-test, with *P*-values shown on the figures.

Since ABA mediates drought stress responses, and the expression levels of *TaPP2C-a5.1* and *TaPP2C-a5.2* were induced by ABA (Fig. 1J-M). In order to explore whether TaPP2C-a5.1 and TaPP2C-a5.2 were also involved in drought stress response, wheat plants were grown in soil under normal conditions for 3 weeks before irrigation was stopped for about 25 d (Fig. 5A, 5E). After 20 d of dehydration, *TaPP2C-a5.1* OE lines (a5.1-OE24 and OE33) began to wilt and could hardly stand upright, while WT plants remained upright (Fig. 5B). After 27 d of drought and 2 d of rehydration, the survival rate of the *TaPP2C-a5.1* OE lines was about 50 %, while approximately 78 % of the WT plants survived (Fig. 5C-D). The *TaPP2C-a5.1* OE lines showed weaker tolerance to drought stress than the WT. However, after 24 d of dehydration, both *TaPP2C-a5.2* OE lines (a5.2-OE2 and OE7) and WT plants exhibited the same degree of wilting and were unable to stand upright (Fig. 5F). When drought stress continued for 7 d and then rehydrated for 2 d, both *TaPP2C-a5.2* OE lines and WT plants showed similar tolerance levels to drought stress (75 % survival rate) (Fig. 5G-H). These data indicate that TaPP2C-a5.1 negatively regulates wheat drought stress tolerance, while TaPP2C-a5.2 does not participate in wheat drought stress response.

## Discussion

ABA not only regulates plant growth and development, but also plant adaptation to biotic and abiotic stress responses [84]. The central role of clade A PP2Cs in ABA signalling has been well-established in Arabidopsis and rice [8,85]. However, the physiological functions and underlying molecular mechanisms, especially at the post-transcriptional level, of clade A PP2Cs in seed dormancy and drought response mediated by ABA signaling remain unclear in wheat. AS is a ubiquitous regulation mechanism of post-transcriptional level of gene expression, which increases transcriptome plasticity and proteome diversity and controls developmental processes and environmental responses in eukaryotes [86]. In this study, we identified that clade A *TaPP2C-a5* underwent AS to produce *TaPP2C-a5.1* and *TaPP2C-a5.2* isoforms in hexaploid wheat (Fig. 1A-C, 6A). *TaPP2C-a5.1* and *TaPP2C-a5.2* transcripts were primarily expressed in seeds and reached peak in the later stages of seed maturation, as well as were induced by ABA treatment. The expression levels of both were also detected during seed germination (Fig. 1F-M). These results suggested that TaPP2C-a5.1 and TaPP2C-a5.2 may be involved in the regulation of wheat seed dormancy, germination and ABA signalling. The previous reports indicated that most AHG1 subfamily members predominantly localized in the nucleus, while all the ABI1 subfamily members localized in both the cytoplasm and nucleus in Arabidopsis and rice [72,87]. Both TaPP2C-a5.1 and TaPP2C-a5.2, belonging to AHG1 subfamily, were also located in the nucleus (Fig. 1N). These results suggested that TaPP2C-a5.2 isoform was identified at both the mRNA and protein levels in hexaploid wheat, further confirming that the *TaPP2C-a5* underwent AS. Furthermore, only *OsPP2C08* full-length transcript was cloned and no other transcripts existed in rice *cv*. Nipponbare (Fig. 1A, 1D), and the Rice Annotation Project database [88]. At present, there are no studies showing that clade A *OsPP2Cs* undergo AS. However, we found that the *TdPP2C-a5* also underwent the same AS as *TaPP2C-a5* in tetraploid wheat *cv*. Luna and confirmed the presence of *TdPP2C-a5.1* and *TdPP2C-a5.2* variants at mRNA level (Fig. 1A, 1E; Fig. S3D-E). Together with the full-length transcriptome data supported the PacBio Iso-seq technologies in sorghum, maize, brachypodium, and foxtail millet, these results strongly indicated that *TaPP2C-a5.2* AS could be probably Triticeae-specific. This seems reasonable because wheat is mainly grown in temperate regions and frequently needs to survive through winter, while, by contrast, the other Poaceae food crops are cultivated during summer and autumn in environmental conditions relatively more humid and warmer than those of wheat. Wheat obviously has better opportunity for seed dormancy, germination, and drought stress responses. The dramatic differences in growth conditions and evolutionary trajectories could explain why the *TaPP2C-a5.2* AS was only detected in the Triticeae species. Among nine clade A *AtPP2Cs*, only *HAB1*, a member of ABI1 subfamily, had been reported to subject to AS, producing HAB1.1 and HAB1.2 isoforms. The *HAB1.2*, containing the third intron, encoded a smaller protein because of a premature stop codon. HAB1.1 and HAB1.2 played opposing roles in ABA-mediated seed germination and post-germination growth arrest, which represented a key mechanism for the turning ABA signalling on and off [11]. Meanwhile, *ZmPP2C26*, belonging to the clade B *ZmPP2Cs* and typically not involved in ABA signaling, underwent alternative exon 1 splicing to produce *ZmPP2C26L* and *ZmPP2C26S* isoforms, and overexpression of both isoforms significantly reduced drought tolerance in *Arabidopsis* and rice [89]. Different from Arabidopsis and maize, *TaPP2C-a5.2*, without 106 bp exon 2, encoded a smaller protein because of a premature stop codon UGA, potentially resulting in a partial catalytic domain (Fig. 1A). These findings suggest that an elaborate and fine regulatory mechanism mediated by clade A PP2Cs at the post-transcriptional level may have been evolved in Triticeae, and lay a foundation for the study of the complex regulatory mechanism of wheat ABA signaling.

DOG1 controls seed dormancy by suppressing the action of specific AHG1/AHG3 phosphatases in Arabidopsis [48,72]. We found that TaDOG1L1 interacted with TaPP2C-a5.1, not TaPP2C-a5.2, potentially affecting the phosphatase activity of TaPP2C-a5.1 (Fig. 2B-C; Fig. S4). This may result in the PVSKs of *TaPP2C-a5.1* transgenic wheat slightly lower than those of *TaPP2C-a5.2* transgenic wheat (Fig. 3A, 3C). There were two canonical interfaces between ABA-PYLs and PP2Cs, one involved the CL2 loop of ABA-PYLs and the PP2Cs active site (Gly and Glu near the active site), and the other consisted of hydrophobic pockets of ABA-PYLs and an invariant Trp in PP2Cs (e.g., Trp^280^ in AHG3, Trp^385^ in HAB1, Trp^300^ in ABI1, Trp^290^ in ABI2, etc.) (Fig. S2). The conserved Trp as ABA sensor is crucial for stabilizing the PYLs-ABA-PP2Cs complex [77,78,90,91]. In Arabidopsis, seed-specific AHG1 was the only clade A PP2C that lacked a conserved Trp site (Fig. S2). AHG1 was not virtually inhibited by PYLs, thereby allowing it to negatively regulate active ABA signaling in seeds, even in the presence of high levels of ABA-PYL complexes [48,92]. Moreover, the mutation of the conserved Trp residue in HAB1 to Ala effectively abolished the inhibitory effect of ABA-dependent PYLs on HAB1 [79]. In the presence of ABA, the TaPP2C-a5.2 did not interact with any TaPYLs, possibly lacking the conserved Trp located in exon 2, while TaPP2C-a5.1 interacted with TaPYLs (Fig. 2F-G; Fig. S2). Both TaPP2C-a5.1 and TaPP2C-a5.2 interacted with class III TaSnRK2.8 (Fig. 2J-K; Fig. S4). These results suggested that TaPP2C-a5 plays an important role in wheat ABA signaling. Overexpression of both *TaPP2C-a5.1* and *TaPP2C-a5.2* reduced seed dormancy levels and led to PHS phenotype in wheat (Fig. 3A, 3C). Overexpression of both *TaPP2C-a5.1* and *TaPP2C-a5.2* also decreased the sensitivity of ABA-inhibited seed germination and post-germination growth in wheat (Fig. 3B, 3E-F, 4A-H). As with most signaling transduction pathways, the ABA response ultimately leads to changes in gene expression [93]. The expression levels of *TaABI5* were decreased in both *TaPP2C-a5.1* and *TaPP2C-a5.2* OE lines (Fig. 3D). These results suggested that both TaPP2C-a5.1 and TaPP2C-a5.2, as negative regulators of ABA signaling, were involved in the regulation of ABA signaling mediated seed dormancy and germination. In addition, overexpression of both *TaPP2C-a9* and *TaPP2C-a10* decreased the sensitivity of seed germination to ABA in Arabidopsis [38,39]. These results suggested that the AHG1 subfamily members may negatively regulate ABA signaling mediated seed germination in wheat. The ABA content is closely related to the seed dormancy level. Compared with WT, the expression levels of *TaABA8′OH1* and *TaABA8′OH2* were decreased and increased, respectively, in both *TaPP2C-a5.1* and *TaPP2C-a5.2* OE lines (Fig. S6C-D), suggesting that the ABA catabolic process may be maintained in homeostasis in *TaPP2C-a5* OE lines. While the expression levels of *TaNCED1* and *TaNCED2* were increased in both *TaPP2C-a5.1* and *TaPP2C-a5.2* OE lines (Fig. S6A-B). Overexpression of *TaPP2C-a5* isoforms promoted turn-off of ABA signaling, which may lead to increased expression levels of *TaNCEDs,* thereby increasing endogenous ABA levels, which interact with other hormone signals to maintain the basic physiological activities of *TaPP2C-a5* OE lines.

**Fig. 6:**
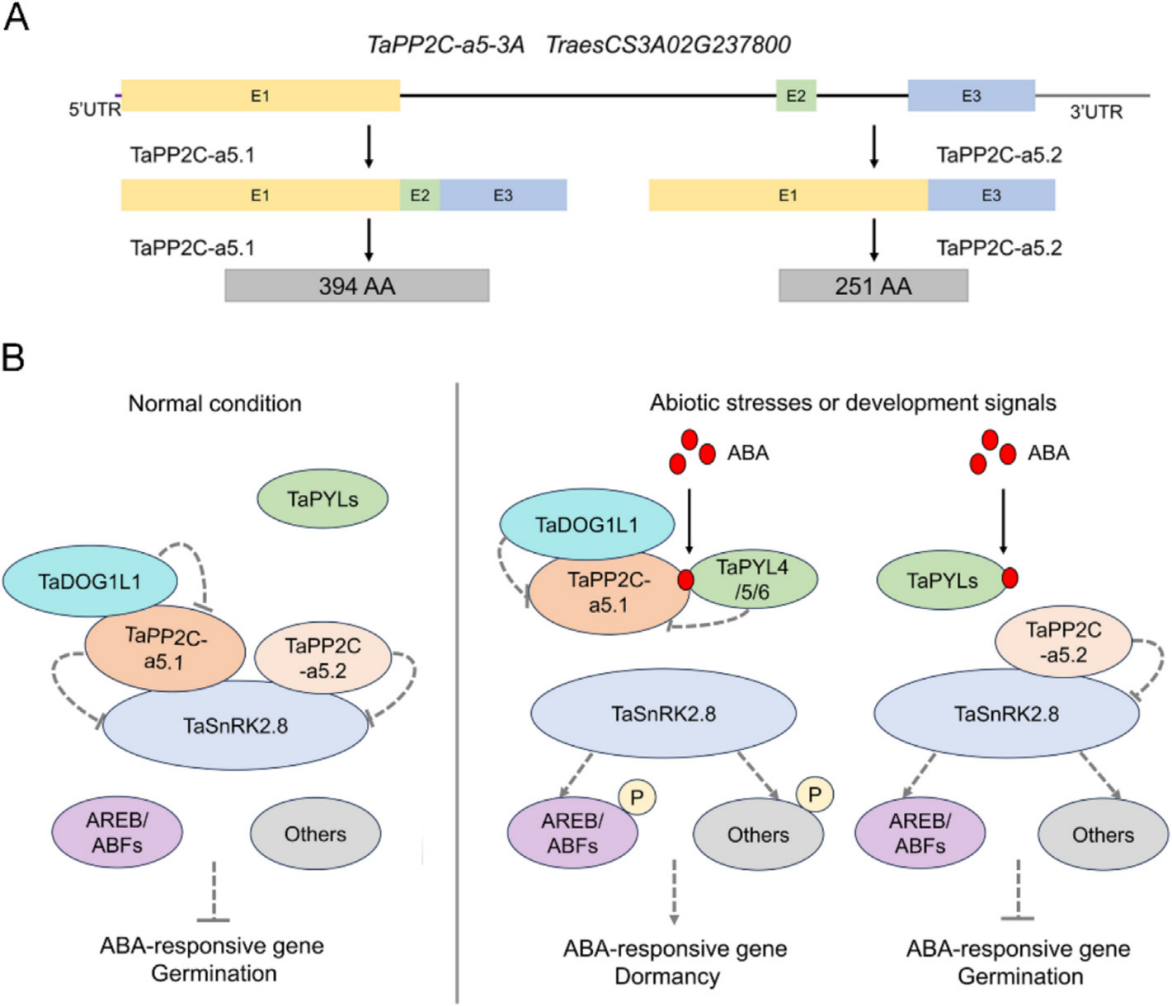
A proposed model showing the TaPP2C-a5 plays an essential role in the DOG1 and ABA pathways to regulate seed dormancy and germination in wheat. (**A**) *TaPP2C-a5* gene undergoes AS to produce *TaPP2C-a5.1* and *TaPP2C-a5.2* isoforms in wheat. (**B**) The TaPP2C-a5.1 and TaPP2C-a5.2 isoforms coordinately regulate ABA signaling mediated seed dormancy and germination in wheat. The DOG1 and ABA pathways are connected at the clade A TaPP2C-a5 to control seed dormancy and germination in wheat. The grey solid lines represent inhibition, and the grey arrows represent activation.

Based on our results, we propose a possible regulatory mechanism that the DOG1 and ABA pathways were connected at the clade A TaPP2C-a5 to control seed dormancy and germination in wheat (Fig. 6B). Under normal growth conditions, both TaPP2C-a5.1 and TaPP2C-a5.2 interacted with TaSnRK2.8 and possibly inhibited its kinase activity to turn off ABA signalling to promote seed germination. While TaDOG1L1, as a positive regulator of seed dormancy [94,95], interacted with TaPP2C-a5.1 and possibly suppressed its action, but not TaPP2C-a5.2. At this time, the presence of TaPP2C-a5.2 may replace TaPP2C-a5.1 as a negative regulator of ABA signaling to maintain normal ABA signal transduction and promote wheat seed germination. In the presence of ABA, ABA bound to TaPYL4/5/6 to promote the interactions with TaPP2C-a5.1 and possibly inhibited its phosphatase activity, which released the kinase activity of TaSnRK2.8 to activate ABA signalling to promote seed dormancy. TaPP2C-a5.2 did not interact with TaDOG1L1 or TaPYLs. At the same time, the presence of TaPP2C-a5.2 may turn off the excessive ABA signaling caused by high levels of ABA accumulated during wheat seed maturation and enhanced by the TaDOG1L1-TaPP2C-a5.1 complex, and to promote seed germination while avoiding adverse effects on the growth and development of wheat. This suggests that a mechanism may have been evolved through AS of clade A *TaPP2C-a5* to fine-tune seed ABA signaling and to maintain homeostasis during seed maturation at the post-transcriptional level in Triticeae. In the future, further phosphatase experiments should be performed to determine the self- and inhibited-phosphatase activity of TaPP2C-a5.1 and TaPP2C-a5.2. The *TaPP2C-a5.1*–*3 × HA* and *TaPP2C-a5.2*–*3 × HA* OE lines should be performed to GST pull-down analyses and the immunoprecipitation-mass spectrometry (IP-MS) to identify TaPYLs interacting with TaPP2C-a5, and to elucidate the mechanism of the TaPYLs-ABA-TaPP2Cs interaction. The PHS-resistant wheat could be obtained by knocking out *TaPP2C-a5.1* and *TaPP2C-a5.2*. Then the complex regulatory mechanisms of TaPP2C-a5 at the post-transcriptional level, which precisely regulated seed dormancy and germination mediated by ABA signaling, should be further analyzed in wheat, in order to provide candidate genes for wheat PHS resistance germplasm. In addition, it is necessary to explore the functions of *TaPP2C-a5* isoforms using population genetics. Wheat seed resources in which *TaPP2C-a5* does not undergo AS, populations with variations in *TaPP2C-a5* isoforms, and populations with significantly different expression levels of *TaPP2C-a5.1* and *TaPP2C-a5.2* should be identified to study the association between AS and phenotype. This will further enhance our understanding of the AS mechanism of *TaPP2C-a5* and the functions of its isoforms, which is significant for wheat breeding programs aiming to improve crop resilience and performance.

The stress response is essential for sessile plants because they have to tolerate environmental challenges. ABA is involved in the regulation of drought stress response [96]. Under drought and exogenous ABA treatment, the biomass of wheat seedlings was increased in the *TaPP2C6* (also called *TaPP2C-a3*) knockout mutant, while overexpression resulted in the opposite phenotype [97]. The TaPP2C-a10 also reduced drought tolerance of transgenic Arabidopsis [38]. We found that TaPP2C-a5.1 negatively regulated drought stress response in wheat (Fig. 5A-D). These results suggest that clade A TaPP2Cs may be negative regulators of drought stress response. Although *TaPP2C-a5.2* was also induced by ABA, its overexpression was not involved in obvious regulation of drought stress response in wheat, which may mainly attribute to the regulation of seed dormancy and germination (Fig. 1L-M, 5E-H). Based on the above results, it is reasonable to speculate that the knockout mutant of *TaPP2C-a5.1* might enhance both PHS resistance and drought stress tolerance in wheat, while the knockout mutants of *TaPP2C-5.2* may enhance PHS resistance, but may not possibly affect drought stress response in wheat. Following this logic and the phenotypes of overexpression lines, seed-specific manipulation of *TaPP2C-a5.2* may provide a novel way to fine-tune seed dormancy and germination and improve PHS resistance in wheat, while ABA signaling-mediated drought stress response remains unaffected. We, thereby, suggest that manipulation of *TaPP2C-a5.2* represents a potential strategy to bypass possible stress-growth trade-off that could be often seen when the ABA signaling components are manipulated. This proposed strategy warrants future investigations.

In humans, AS is often tightly regulated in a cell-specific, tissue-specific, or cell response to environmental factors dependent manners [[98], [99], [100]]. Understanding the AS mechanism requires knowledge of the regulatory networks of protein-RNA and protein–protein interactions. Through the study of individual transcripts and genome-wide methods, significant progress has been made in understanding the regulation mechanisms of AS of pre-mRNA. Splicing site selection usually depends on the *cis*- or *trans*-regulatory elements and the activities of splicing factors, such as exonic splicing silencer (ESS), exonic splicing enhancer (ESE), intronic splicing silencer (ISS), intronic splicing enhancer (ISE), SR protein, hnRNPs, NOVA and RBM [[101], [102], [103]]. HsRBM25, a RNA-recognition motif-containing protein, regulates the 5′ splicing site selection of exon 2 of *Bcl-x* pre-mRNA, producing the opposite functional Bcl-x(L) and Bcl-x(s) isoforms during apoptosis [104]. HsRBM39 regulates exon 2 skipping of *RFX1* pre-mRNA, producing the N-terminal truncated RFX1, which loses the transcriptional inhibitory activity on oncogenic collagen genes [105]. The splicing factor AGGF1 promotes exon 3 skipping of *SRSF6* pre-mRNA to produce the full-length SRSF6, which is important for cell proliferation and migration [106]. In *Arabidopsis*, RBM25 directly binds to the 5′ splice site of intron 3 of *HAB1* pre-mRNA, and regulates AS and molecular diversity of *HAB1* [11]. Subsequently, RBM25 can control pre-mRNA splicing of many genes, and transcriptional levels and post-translational expression of *RBM25* are regulated by ABA [60]. AtGRP20, encoding a non-classical RNA-binding protein, regulates splicing of micro-exon and small-exon of response and development genes [107]. Compared to humans, the specialized splicing factors and transcript-specific regulation mechanisms of essential genes remain largely unknown in plants, especially in crops with complex genomes. The spatiotemporal expression specificity of *TaPP2C-a5.2*, losing the 106 bp exon 2, provides a good example for identifying the splicing factors and *cis*-regulatory elements that control the specific biological processes in Triticeae. The exploration of AS mechanisms of *TaPP2C-a5* is of great importance for understanding the regulation of ABA signalling and drought stress response, and can be further studied in the future.

## Conclusion

In summary, *TaPP2C-a5* gene underwent AS to produce two transcripts and was involved in the regulation of seed dormancy and germination as well as drought stress response mediated by ABA signaling pathway. Both TaPP2C-a5.1 and TaPP2C-a5.2 negatively regulated wheat seed dormancy levels, and promoted seed germination. In addition, both TaPP2C-a5.1 and TaPP2C-a5.2 negatively regulated ABA-mediated seed germination as well as post-germination developmental arrest in wheat. TaPP2C-a5.1 negatively regulated drought stress response, while TaPP2C-a5.2 did not involve in drought stress response in wheat. Our work reveals the important role of TaPP2C-a5 AS in wheat ABA signaling. In the future, exploring potential regulatory factors involved in *TaPP2C-a5* AS will be of great importance for understanding the complex regulation of ABA signaling and stress responses in Triticeae. The acquisition of knockout mutants of *TaPP2C-a5* isoforms can provide candidate genes for wheat PHS resistance and drought stress resistance germplasm. In addition, it is necessary to study the association between *TaPP2C-a5* AS and phenotype using population genetics, which is significant for wheat breeding programs.

## Compliance with ethics requirements

*This article does not contain any studies with human or animal subjects.*

## CRediT authorship contribution statement

**Qian Zhang:** Methodology, Investigation, Data curation, Writing – original draft. **Xiaofen Yu:** Conceptualization. **Ya’nan Wu:** Methodology, Formal analysis, Validation. **Ruibin Wang:** Visualization. **Yufan Zhang:** Data curation. **Fu Shi:** Investigation. **Hongyan Zhao:** Methodology. **Puju Yu:** Software, Validation. **Yuesheng Wang:** Investigation, Resources, Supervision. **Mingjie Chen:** Resources, Supervision. **Junli Chang:** Resources, Supervision. **Yin Li:** Conceptualization, Resources, Supervision, Funding acquisition, Writing – review & editing. **Guangyuan He:** Conceptualization, Resources, Supervision, Project administration, Funding acquisition, Writing – review & editing. **Guangxiao Yang:** Conceptualization, Resources, Supervision, Project administration, Funding acquisition, Writing – review & editing.

## Declaration of competing interest

*The authors declare that they have no known competing financial interests or personal relationships that could have appeared to influence the work reported in this paper.*

## Data Availability

Data will be made available on request.
