# Supplementary material for: TaPP2C-a5 fine-tunes wheat seed dormancy and germination with a Triticeae-specific, alternatively spliced transcript
Source: J Adv Res. 2025 May 8;80:79–93. doi: 10.1016/j.jare.2025.05.007 (PMC12869235; doi:10.1016/j.jare.2025.05.007)
Supplement: Supplementary Data 1 [file mmc1.docx]

Supplementary Information for

**TaPP2C-a5 fine-tunes wheat seed dormancy and germination with a Triticeae-specific, alternatively spliced transcript**

Qian Zhang^a,1^, Xiaofen Yu^b,1^, Ya’nan Wu^a,1^, Ruibin Wang^a^, Yufan Zhang^a^, Fu Shi^a^, Hongyan Zhao^a^, Puju Yu^a^, Yuesheng Wang^a^, Mingjie Chen^a^, Junli Chang^a^, Yin Li^a,*^, Guangyuan He^a,*^, Guangxiao Yang^a,*^

^a^ The Genetic Engineering International Cooperation Base of Chinese Ministry of Science and Technology, Key Laboratory of Molecular Biophysics of Chinese Ministry of Education, College of Life Science and Technology, Huazhong University of Science and Technology, Wuhan 430074, China

^b^ Key Laboratory of Plant Germplasm Enhancement and Specialty Agriculture, Wuhan Botanical Garden, Innovative Academy of Seed Design, Chinese Academy of Sciences, Wuhan 430074, China

^1^ These authors contributed equally to this work

^*^ Corresponding authors: Yin Li ([yinli2021@hust.edu.cn](mailto:yinli2021@hust.edu.cn)), Guangyuan He (hegy@hust.edu.cn), Guangxiao Yang (ygx@hust.edu.cn)

Tel: +86-27-87792271

Fax: +86-27-87792272

This file contains the following supporting information.

**Figures**

**Fig. S1.** Expression analysis of tissue-specificity and drought stress response in class A *TaPP2Cs*.

**Fig. S2.** Sequence alignment analysis of the clade A AtPP2Cs and TaPP2C-a5.

**Fig. S3.** Analyses on the homologous genes of *TaPP2C-a5*.

**Fig. S4.** The BiFC analysis of TaPP2C-a5 with TaDOG1L1 and TaSnRK2.8.

**Fig. S5.** Production of the transgenic wheat plants and the expression analyses of the transgenic *TaPP2C-a5.1* and *TaPP2C-a5.2*.

**Fig. S6.** Analysis of expression levels of ABA biosynthesis and catabolism genes.

**Tables**

**Table S1.** Primers used in the present study.

**Table S2.** The amino acid and CDS sequences of TaPP2C-a5.

**Table S3.** The alternative splicing of homologous genes of *TaPP2C-a5*.

**Table S4.** The gDNA and candidate transcript sequences of *HORVU3Hr1G059170* gene in barley.

**Table S5.** The gDNA and candidate transcript sequences of *AeCom.PI551049.r1.3MG022460* gene in *Aegilops comosa*.

**Table S6.** Two-way ANOVA analysis results for the comparison of germination rates between WT and OE lines of wheat.

**Table S7.** Two-way ANOVA analysis results for the comparison of shoot and root lengths between WT and OE lines of wheat.


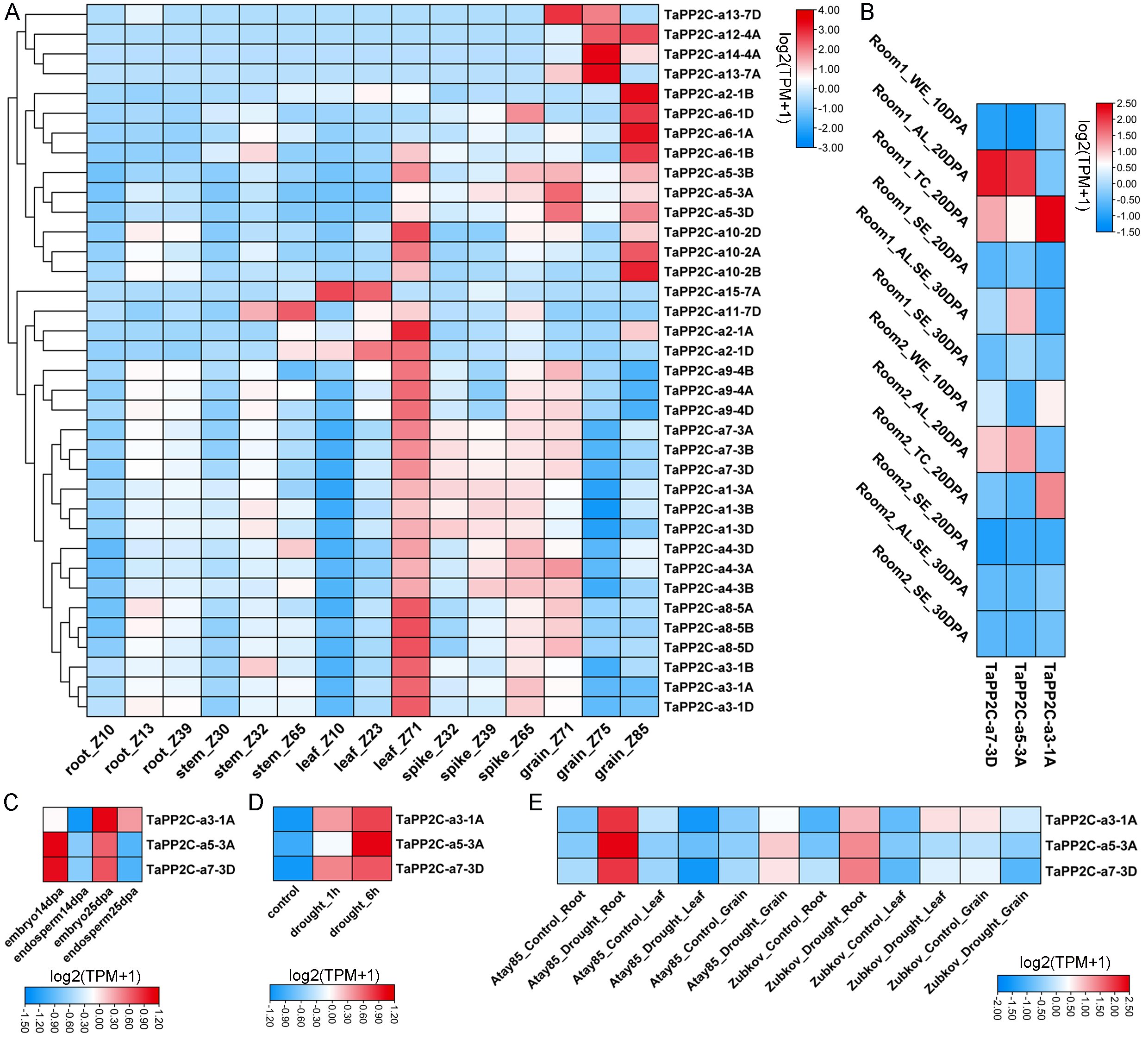


**Fig. S1.** Expression analysis of tissue-specificity and drought stress response in class A *TaPP2Cs*. The expression profiles of class A *TaPP2Cs* were shown in the heatmap in different conditions: (**A**) across wheat developmental stages (root, stem, leaf, spike and grain), (**B**) in different cell types of developing wheat grain, (**C**) in the embryo and endosperm of developing wheat grain, (**D**) in response to drought stress and (**E**) in various tissues under drought stress. Root_Z10 (seedling stage), Z13 (three leaf stage) and Z39 (flag leaf stage); Stem_Z30 (1 cm spike stage), Z32 (two-node stage) and Z65 (anthesis stages); Leaf_Z10 (seedling stage), Z23 (flag leaf at tillering) and Z71 (2 dpa stages); Spike_Z32 (two-node stage), Z39 (flag leaf stage) and Z65 (anthesis stages); Grain_Z71 (2 dpa stages), Z75 (14 dpa stages) and Z85 (30 dpa stages). WE: whole endosperm; SE: starchy endosperm; AL: aleurone layer; TC: transfer cells. Red and blue indicate relative higher and lower expression levels. The gene expression levels were quantified using log_2_(TPM + 1).

**

**

Fig. S2. Sequence alignment analysis of the clade A AtPP2Cs and TaPP2C-a5. Red box indicates the conserved Trp (e.g., Trp^280^ in AHG3, Trp^385^ in HAB1, Trp^300^ in ABI1, Trp^290^ in ABI2, etc.) sites between clade A PP2Cs. Blue arrow indicates the conserved Glu (e.g., Glu^142^ in ABI1, Glu^203^ in HAB1, etc.) and Gly (e.g., Gly^180^ in ABI1, Gly^246^ in HAB1, etc.) sites around clade A PP2Cs active site. The blue line represents the phosphatase domain of TaPP2C-a5.

**
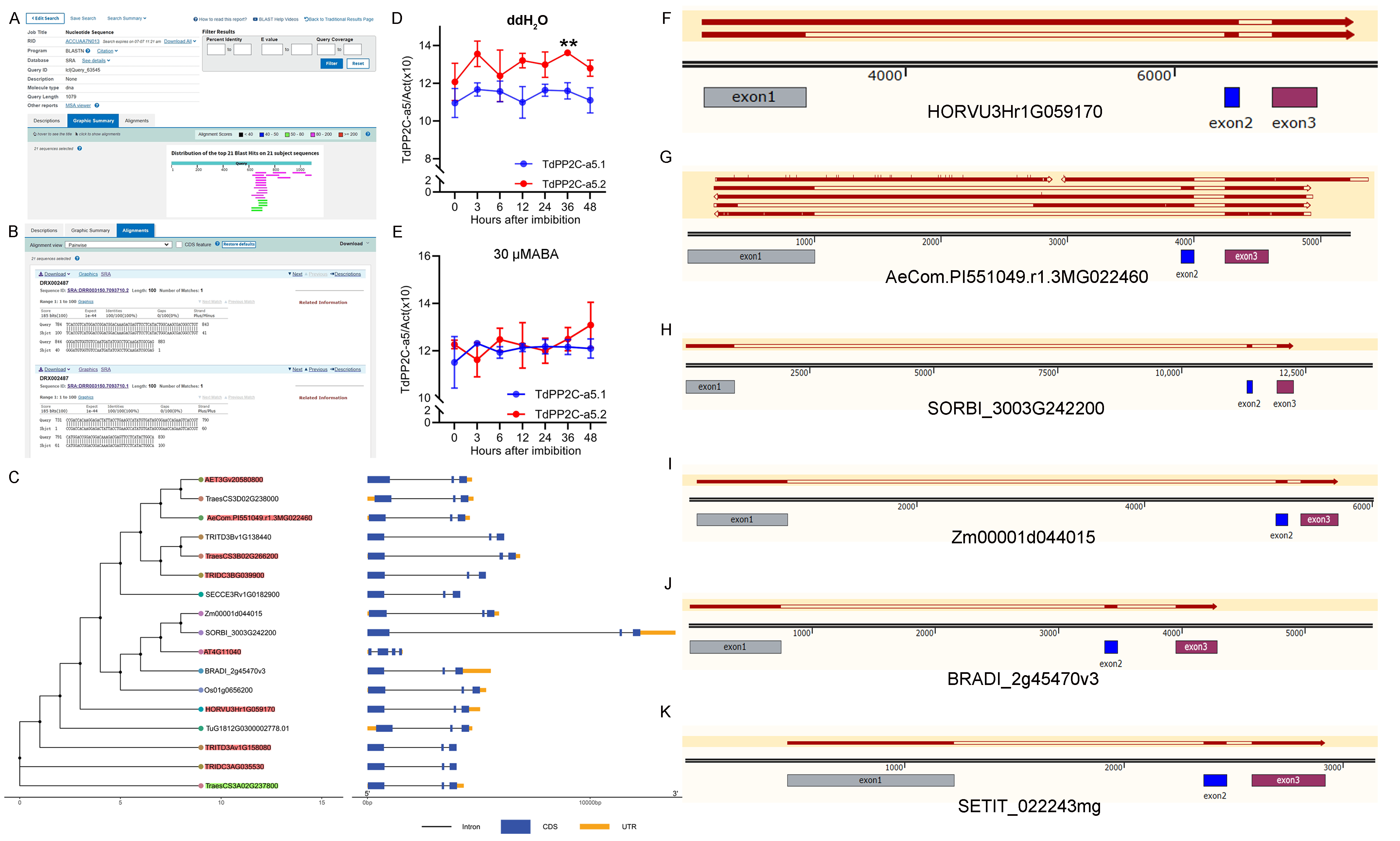
**

Fig. S3. Analyses on the homologous genes of *TaPP2C-a5*. (**A**, **B**) Images confirming the presence of the *TaPP2C-a5.2* transcript. Data are from SRA database in NCBI (https://blast.ncbi.nlm.nih.gov/Blast.cgi). (**C**) The gene structures homologous genes of *TaPP2C-a5* in different species. AT: *Arabidopsis thaliana*; Os: *Oryza sativa*; Zm: *Zea mays*; SORBI: *Sorghum bicolor*; BRIAD: *Brachypodium distachyon*; HORVU: *Hordeum vulgare*; SECCE: *Secale cereale*; Tu: *Triticum urartu*; AET: *Aegilops tauschii*; Aecom: *Aegilops comosa*; TRITD: *Triticum turgidum*; TRIDC: *Triticum dicoccoides*; Traes: *Triticum aestivum*. Data are obtained from Triticeae Gene Tribe (TGT) database (http://wheat.cau.edu.cn/TGT/). The green represents *TaPP2C-a5-3A* gene. The red indicates that *TaPP2C-a5*-A homologous genes may undergo alternative splicing speculating from the ensembl plants database (http://plants.ensembl.org/index.html) and PacBio Iso-seq data. (**D**, **E**) qRT-PCR analysis of *TdPP2C-a5.1* and *TdPP2C-a5.2* expression patterns during seed imbibition time points with (**E**) or without 30 μM ABA (**D**). Statistical differences of expression levels between transcripts for the same time point were determined by Student’s *t*-test (** *P* < 0.01). The expression data were presented as means ± standard error (S.E.), with the sample size and related information described in Figure 1’s legend. (**F-K**) The annotation of gene exon-intron structures (shown in colored boxes) and annotated transcripts (shown in dark red) of the *TaPP2C-a5* orthologs, *HORVU3Hr1G059170* in *Hordeum vulgare* (**F**), *AeCom.PI551049.r1.3MG022460* in *Aegilops comosa* (**G**), *SORBI_3003G242200* in *Sorghum bicolor* (**H**), *Zm00001d044015* in *Zea mays* (**I**), *BRADI_2g45470v3* in *Brachypodium distachyon* (**J**), and *SETIT_022243mg* in *Setaria italica* (**K**).

**
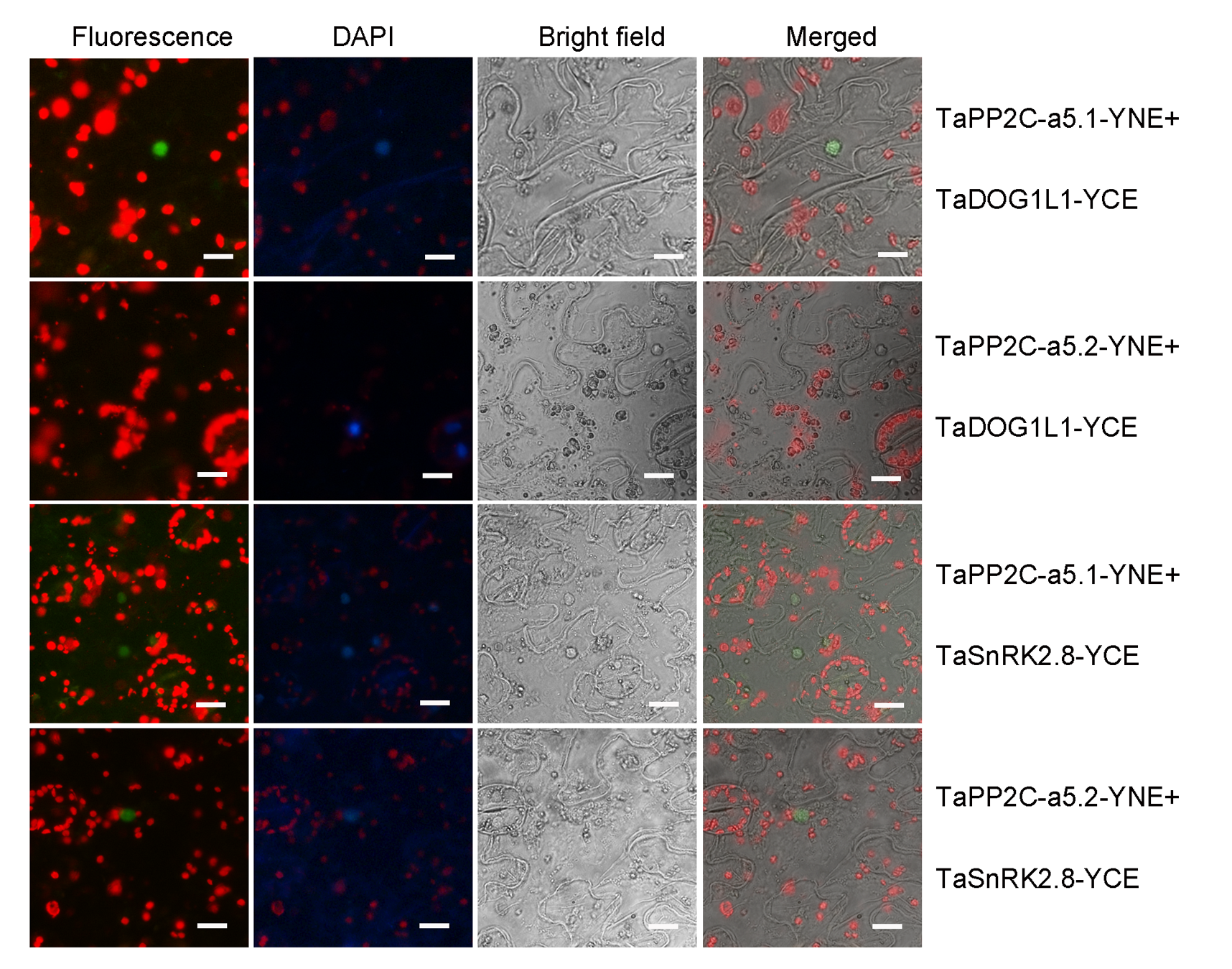
**

**Fig. S4.** The BiFC analysis of TaPP2C-a5 with TaDOG1L1 and TaSnRK2.8. Interactions between TaPP2C-a5 isoforms and TaDOG1L1 as well as TaSnRK2.8 in BiFC assay. The TaPP2C-a5.1-YNE or TaPP2C-a5.2-YNE were co-transformed into tobacco leaf cells with TaDOG1L1-YCE or TaSnRK2.8-YCE, respectively. At 48 h after transformation, YFP signals were observed. The cell nucleus was stained with 4′,6-diamidino-2-phenylindole (DAPI). Scale bar represents 20 μm. The BiFC experiments were repeated three times with similar results, and representative images were presented.

**
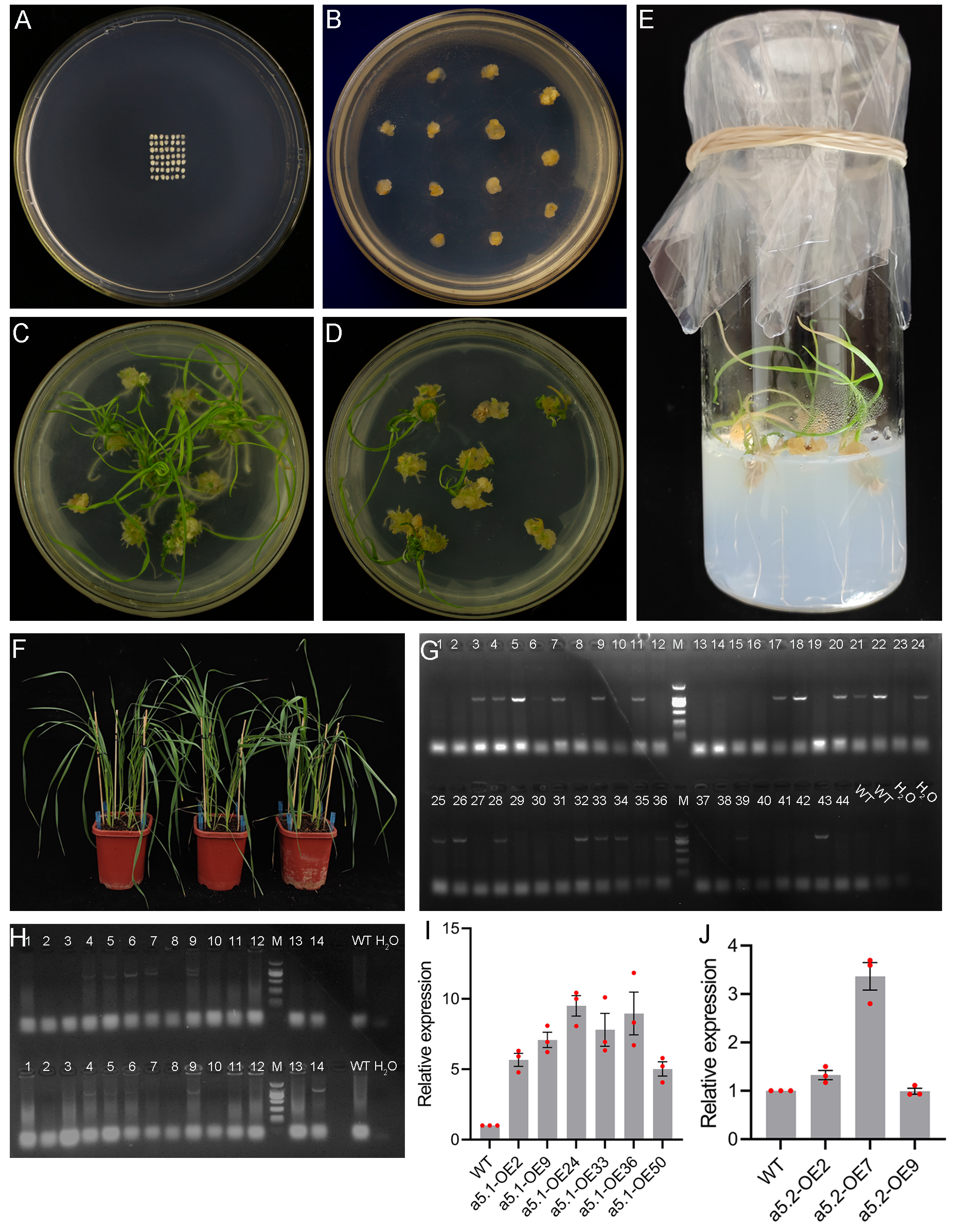
**

**Fig. S5.** Production of the transgenic wheat plants and the expression analyses of the transgenic *TaPP2C-a5.1* and *TaPP2C-a5.2*. (**A**) The intact young embryos removed from immature wheat seeds. (**B**) Young embryos bombarded with gene gun were transferred to induction medium to induce callus. (**C**) Callus were transferred to differentiation medium for differentiation culture. (**D**) Callus were transferred to differentiation medium containing glufosinate-ammonium for screening culture. (**E**) The seedlings from differentiation culture were transferred to rooting medium for rooting culture. (**F**) The rooted wheat seedlings were transplanted to nutrient soil for culture. (**G**, **H**) These are representative agarose gel electrophoresis maps of *TaPP2C-a5.1* (**G**) and *TaPP2C-a5.2* (**H**) transgenic wheat of T_1_ generation, respectively. The primer was GUS-2F/2R, the target band size was 1053 bp, and WT and H_2_O were used as negative controls. (**I**, **J**) The wheat seeds at 28 dpa from WT, *TaPP2C-a5.1* (**I**) and *TaPP2C-a5.2* (**J**) OE lines were used for qPCR-based expression assay, respectively. *TaActin* was used as the internal reference gene. The expression data were presented as means ± S.E. of three biological replicates, with each replicate meaning the pooled sample of six seeds per WT or OE line.


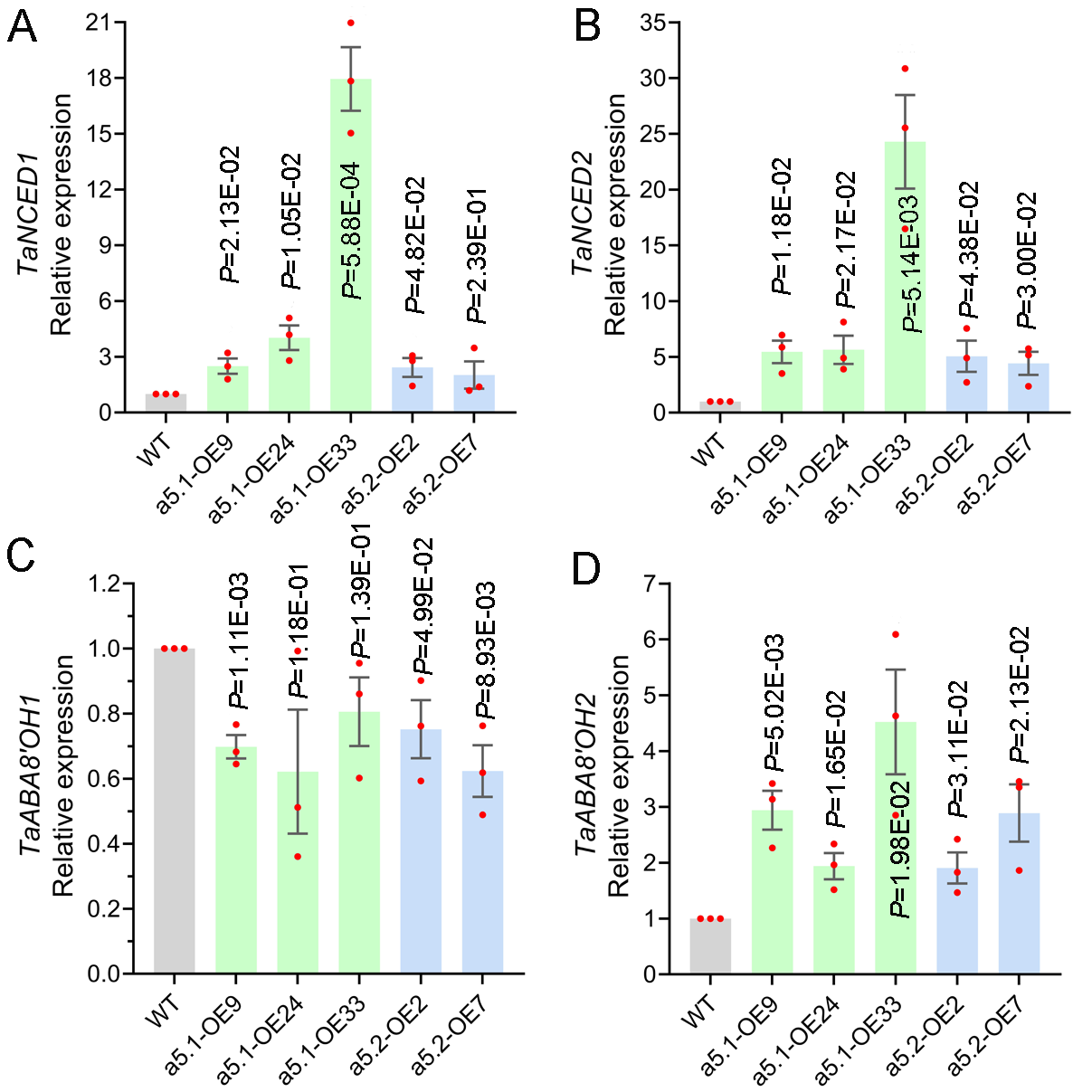


**Fig. S6.** Analysis of expression levels of ABA biosynthesis and catabolism genes. The qRT-PCR analysis of *TaNCED1* (**A**), *TaNCED2* (**B**), *TaABA8'OH1* (**C**) and *TaABA8'OH2* (**D**) expression level in germinated seeds of *TaPP2C-a5.1*, *TaPP2C-a5.2* OE lines and WT. The expression levels were presented as means ± S.E. of three independent replicates, with the sample size and related information described in Figure 3’s legend. Differences in gene expression levels were determined between WT and OE lines by using Student’s *t*-test, with *P*-values indicated.
